# Using Feature‐Assisted Machine Learning Algorithms to Boost Polarity in Lead‐Free Multicomponent Niobate Alloys for High‐Performance Ferroelectrics

**DOI:** 10.1002/advs.202104569

**Published:** 2022-03-06

**Authors:** Seung‐Hyun Victor Oh, Woohyun Hwang, Kwangrae Kim, Ji‐Hwan Lee, Aloysius Soon

**Affiliations:** ^1^ Department of Materials Science and Engineering and Center for Artificial Synesthesia Materials Yonsei University Seoul 03722 Republic of Korea

**Keywords:** computational materials, feature engineering, ferroelectric oxides, machine‐learning algorithms, multicomponent alloys

## Abstract

To expand the unchartered materials space of lead‐free ferroelectrics for smart digital technologies, tuning their compositional complexity via multicomponent alloying allows access to enhanced polar properties. The role of isovalent A‐site in binary potassium niobate alloys, (K,A)NbO_3_ using first‐principles calculations is investigated. Specifically, various alloy compositions of (K,A)NbO_3_ are considered and their mixing thermodynamics and associated polar properties are examined. To establish structure‐property design rules for high‐performance ferroelectrics, the sure independence screening sparsifying operator (SISSO) method is employed to extract key features to explain the A‐site driven polarization in (K,A)NbO_3_. Using a new metric of agreement via feature‐assisted regression and classification, the SISSO model is further extended to predict A‐site driven polarization in multicomponent systems as a function of alloy composition, reducing the prediction errors to less than 1%. With the machine learning model outlined in this work, a polarity‐composition map is established to aid the development of new multicomponent lead‐free polar oxides which can offer up to 25% boosting in A‐site driven polarization and achieving more than 150% of the total polarization in pristine KNbO_3_. This study offers a design‐based rational route to develop lead‐free multicomponent ferroelectric oxides for niche information technologies.

## Graphical abstract

Using a new metric of agreement via feature‐assisted regression and classification, a machine learning‐based approach to construct an accurate and feature‐derived polarity‐composition map to aid experimentalists in their search for high‐performance lead‐free multicomponent ferroelectrics in the unchartered vast chemical space of KNbO_3_‐based alloys is proposed.

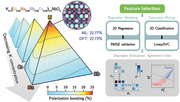

## 1 Introduction

Ferroelectrics are an important class of functional materials used in a variety of information and digital technologies including multilayer ceramic capacitors,^[^ ^1^ ^]^ ferroelectric random access memory,^[^ ^2^ ^]^ ferroelectric photovoltaic device,^[^ ^3^ ^]^ or energy converters.^[^ ^4^, ^5^ ^]^ In addition, ferroelectric materials also inherently exhibit piezoelectricity and pyroelectricity,^[^ ^6^ ^]^ which makes them suitable for many related technological applications. Given their broad applicabilities to many modern technologies, high‐performance ferroelectric materials with bolstered spontaneous polarizations are in high demand.

Notably, due to increasing environmental concerns, lead‐free ferroelectrics^[^ ^6^, ^7^, ^8^ ^]^ have been in the limelight of eco‐friendly materials design and development. One of the more promising ferroelectric oxides, potassium niobate (KNbO_3_; from now also referred to as KNO) and its alloys are widely investigated for its high tunability of physico‐chemical properties via multi‐cation substitutions, and this family of KNO‐based alloys has definitely extended with time.

Specifically, (K,Na)NbO_3_ (or in short, KNN) alloys, at various compositions, are well‐known emerging functional ferroelectrics due to their high Curie temperature, *T*_c_ along with its attractive electromechanical properties.^[^ ^6^, ^9^, ^10^, ^11^, ^12^ ^]^ In this regard, the successful tuning of ferroelectric properties based on A‐site substitution has set a strong platform for multicomponent A‐site engineering of KNO for enhanced/boosted ferroelectric and piezoelectric performance.^[^ ^10^, ^13^, ^14^, ^15^, ^16^, ^17^ ^]^

In the same vein of thought, experiments on the (K,Li,Na)NbO_3_ ternary alloy system have shown a promising enhancement of electromechanical properties.^[^ ^13^, ^14^ ^]^ Furthermore, other chemical variants of KNO‐based alloys, such as (K,Na,Rb)NbO_3_ and (K,Na,Cs)NbO_3_, have also been investigated for their improved single crystal growth via balancing the ionic radius differences between the co‐dopants.^[^ ^15^, ^16^ ^]^ In addition, it has also been proposed that the multicomponent solubility limit in these “high entropy ceramic alloys” may be even raised further (i.e., potentially higher than what has been reported so far) due to the multicomponent entropy stabilizing effect.^[^ ^18^ ^]^

In spite of all these advantages and the notable interest and importance in developing functional lead‐free ferroelectrics, an atomistic design rule for the precise engineering of these multicomponent polar materials is very much lacking. High‐throughput experiments are often plagued with synthesis challenges and simple first‐principles models alone are deemed inadequate for these highly complex multicomponent alloys.

To address this pressing problem, many studies are now relying on the use of big data‐driven machine learning (ML) algorithms to search the vast chemical space to predict key material properties and have shown promising results. For instance, Tian et al.^[^ ^19^ ^]^ have very recently employed active learning and accelerated search from a high‐dimensional virtual space of multicomponent phase diagrams for BaTiO_3_‐based ferroelectrics. However, like for many materials science and engineering problems, the performance (i.e., the error and efficiency) of these classical ML methods is highly sensitive to the size of available data, and usually very large sets of data are needed for accurate and efficient training and prediction.^[^ ^20^, ^21^ ^]^

In contrast to the aforementioned conventional ML approaches,^[^ ^22^, ^23^ ^]^ the recently developed sure independence screening sparsifying operator (SISSO)^[^ ^24^ ^]^ is a very promising method that helps in the identification of the best physically interpretable descriptor of a target property. By searching extensive nonlinear feature spaces generated via a combination of algebraic/functional operations recursively, SISSO is able to extract effective descriptors for even relatively sparse data.^[^ ^25^, ^26^ ^]^ Therefore, with this cutting‐edge sparse data‐driven technique, one may now derive simple (yet accurate) physically interpretable ML models for the prediction of complex multicomponent ferroelectric alloys.

In this work, using first‐principles density‐functional perturbation theory (DFPT) calculations and an improved SISSO ML model, we aim to examine systematically the structural, thermodynamic, and polar properties of the so‐called binary KNO alloys by varying the A‐site replacement with a gradual substitution of K with A (where A = Li, Na, Rb, and Cs). To illustrate our coupled DFPT+SISSO ML model, we will focus on the polarization boosting (denoted as $\hat{P}$ in this work) in these binary KNO alloys by the mixing of A‐site cations and establish a simple and physically intuitive descriptor for the prediction and classification of the $\hat{P}$ values. By leveraging that this descriptor only contains the atomic primary features of constituent species, we will then proceed to build an improved ML model (using a new metric of agreement) for multicomponent KNO‐based alloys and validate this against DFPT calculations, the well‐known Vegard's relation, and the conventional SISSO model. Last, we will provide an accurate and feature‐derived polarity‐composition map to aid experimentalists in their search for high performance multicomponent ferroelectrics in the unchartered vast chemical space of KNO‐based alloys.

## 2 Results and Discussion

### 2.1 Solid Solutions: Crystal Structures and Stability Predictions

Potassium niobate (KNbO_3_; shorthanded as KNO) undergoes a series of polymorphic phase transitions to lower symmetry phases with decreasing temperature. The most commonly reported polymorphic phases are the cubic ($Pm\bar{3}m$), tetragonal (*P*4*mm*), orthorhombic (*Amm*2), rhombohedral (*R*3*m*) crystals.^[^ ^27^ ^]^ In this work, we have limited our investigation to only the tetragonal and orthorhombic phases since these are the experimentally well‐known phases known to exist at room temperature (300 K) and above.^[^ ^27^, ^28^ ^]^ Here, the calculated KNO lattice constants agree with available experimental lattice parameters^[^ ^27^, ^29^, ^30^ ^]^ (to within 1% as presented in Table S1, Supporting Information).

**Figure 1:**
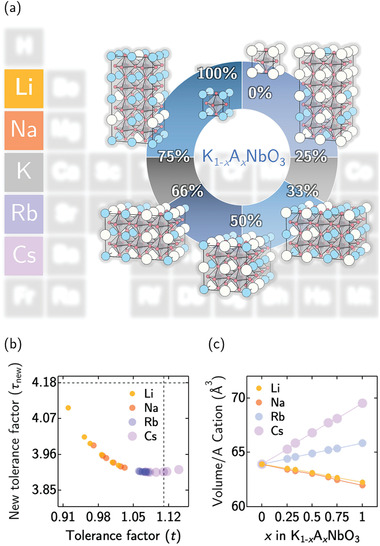
a) Atomic structural models of K_1 − *x*_A_*x*_NbO_3_ (where A = Li, Na, Rb, and Cs) for *x* = 0.00, 0.25, 0.33, 0.50, 0.66, 0.75, and 1.00. The atoms of K, A, Nb, and O are depicted by white, blue, gray, and red spheres, respectively. b) The calculated Goldschmidt (*t*) and new (τ_new_) tolerance factors of K_1 − *x*_A_*x*_NbO_3_ weighted by the atomic size of the A‐site cations. c) The A‐site cation‐normalized volume of K_1 − *x*_A_*x*_NbO_3_ as a function of *x* composition. For (b) and (c), the markers for the corresponding A‐site cations are labeled following the color scheme in legend.

To engineer and derive new properties from KNO, it is common to replace a certain percentage of the K atoms (at the A‐site in KNO) with isovalent Group 1 alkali metals.^[^ ^13^, ^14^, ^15^, ^16^ ^]^ Using the special quasi‐random structure (SQS) approach, the optimized atomistic models of the idealized solid solution K_1 − *x*_A_*x*_NbO_3_ (where A = Li, Na, Rb, and Cs) alloys are shown in **Figure** **1**a. To assess the formability of these alloyed perovskites, simply using the elemental information of Shannon ionic radii^[^ ^31^ ^]^ (where *r*_Li_ < *r*_Na_ < *r*_K_ < *r*_Rb_ < *r*_Cs_; cf. Table S3, Supporting Information) and formal charges (*n*_A_ where a formal charge of +1 is assumed), we calculate and present the conventional Goldschmidt tolerance factor,^[^ ^32^ ^]^ $t=\frac{r_{A}+r_{O}}{\sqrt{2}\left(r_{B}+r_{O}\right)}$, and the new tolerance factor,^[^ ^33^ ^]^ $\tau_{\mathrm{new}}=\frac{r_{X}}{r_{B}}-n_{A}\left(n_{A}-\frac{r_{A}/r_{B}}{\ln(r_{A}/r_{B})}\right)$. More details on the Goldschmidt and new tolerance factors can be found in the Supporting Information.

In Figure 1b, we find that most of the solid solutions are formable, with the exception of pristine CsNbO_3_, which *t* is higher than the cutoff tolerance of *t* = 1.11 (as shown by the vertical dotted line). This instability can be attributed to the large Cs cationic size and formability is improved as the composition of K cations increases between 0.76 < *t* < 1.11.^[^ ^34^ ^]^ In addition, taking τ_new_ < 4.18 as the suggested cutoff limit for formability, we conclude that all proposed K_1 − *x*_A_*x*_NbO_3_ alloy structures are formable. Additional thermodynamic (including configuration entropy considerations^[^ ^35^ ^]^) analysis in Figure S2, Supporting Information corroborates with our findings. We caution that constraining our investigations to the parent tetragonal and orthorhombic phases of KNO is motivated from experimental reports^[^ ^27^, ^28^ ^]^ and the possibility of secondary phase segregation and associated defects are not included.

Besides their formability, we have also examined the impact of cationic exchange on the volume changes (which is known to influence the polar properties of ferroelectrics).^[^ ^36^ ^]^ In Figure 1c, we plot the normalized volume (per A‐site cation) as a function of the compositional changes for the substituents Li, Na, Rb, and Cs. As clearly shown, for these binary K_1 − *x*_A_*x*_NbO_3_ alloys, linear dependencies of the normalized volume with compositional changes nicely reflect the well‐known Vegard's relation^[^ ^35^, ^37^ ^]^ commonly reported for alloys and solid solutions. To be more specific, in case of K_1 − *x*_Li_*x*_NbO_3_ (and K_1 − *x*_Na_*x*_NbO_3_), the normalized volumes decrease with increasing concentrations of Li (and Na). This can be rationalized by the smaller cationic sizes of Li and Na when compared to that of K. Conversely, the normalized volumes of K_1 − *x*_Rb_*x*_NbO_3_ and (K_1 − *x*_Cs_*x*_NbO_3_) increases with increasing concentrations of Rb (and Cs) where now the much larger cationic sizes of Rb and Cs become visible.

In fact, besides the normalized volume, we find a linear dependency for the lattice constants as a function of composition as well (cf. Figure S3, Supporting Information). In a nutshell, the structural properties of binary K_1 − *x*_A_*x*_NbO_3_ alloys via the alkali metal cation‐exchange method can be reasonably justified by the well‐known Vegard's relation.

### 2.2 Trends in Spontaneous Polarization

After addressing the formability of these KNO‐based alloys, we turn our attention to examine the impact of isovalent cation‐exchange on their spontaneous polarization (*P*_s_). The *P*_s_ value for pristine tetragonal and orthorhombic KNO is calculated to be 36.4 and 41.2 μCcm^−2^, respectively. This agrees well with the previously reported experimental value of 37 μCcm^−2^ for tetragonal KNO^[^ ^29^ ^]^ and 41 μCcm^−2^ for orthorhombic KNO.^[^ ^38^ ^]^ The small deviations here are attributed to possible thermal effects where the reported experimental values were obtained at room temperature while our theoretical calculations are performed for 0 K.

By definition (cf. Equation (11)), *P*_s_ is strongly correlated to individual atomic displacements and their associated local electron density fluctuations. On this note, the total *P*_s_ can be decomposed into partial *P*_s_ (for a specific atom or a collective group of atoms). For instance, in this work, it makes sense to inspect the contributions of the A‐site cation and NbO_6_ octahedron (where both the B‐site Nb cation and surrounding O anions operates collectively). It has been previously shown that for the pristine KNO system, K contributes minimally to the total *P*_s_ while the major contribution comes from the NbO_6_ octahedrons.^[^ ^39^, ^40^ ^]^ This suppression of the polarization response of K reflects the strong ionic interactions between K and O ions.^[^ ^39^ ^]^

**Figure 2:**
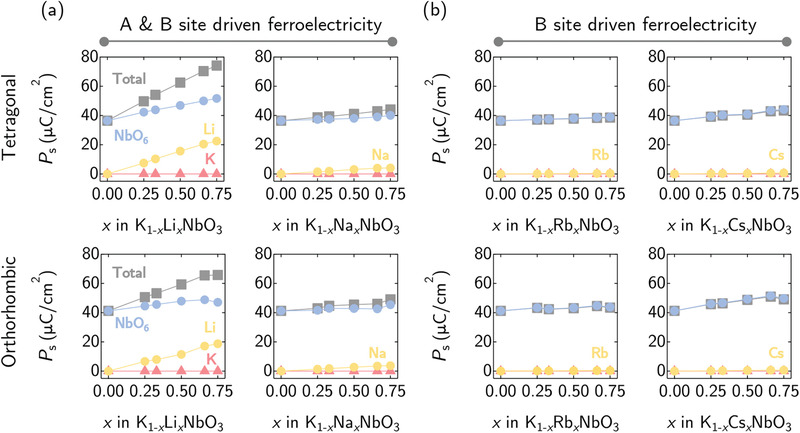
Spontaneous polarization, *P*_s_ of tetragonal (upper panel) and orthorhombic (lower panel) K_1 − *x*_A_*x*_NbO_3_ (where A = Li, Na, Rb, and Cs) as a function of A‐site cation concentration, *x*. To illustrate the site‐specific contributions to *P*_s_, the A‐ and B‐site driven polarization is categorized in (a), while that due predominantly to the B‐site (i.e., due to the NbO_6_ octahedron distortions) in (b). Data points for the total *P*_s_, B‐site cations' contribution, K ion's contribution, and A‐site cations' contribution are represented by gray, blue, red, and yellow markers, respectively.

In **Figure** **2**, we plot the total and partial *P*_s_ (due to the A‐site cations and the NbO_6_ octahedrons) for the KNO‐based alloys in the tetragonal (upper panels) and the orthorhombic (lower panels) phases. We note that the general trends for both polymorphic phases are very similar. In Figure 2a, for K_1 − *x*_Li_*x*_NbO_3_ and K_1 − *x*_Na_*x*_NbO_3_ alloys, the *P*_s_ is clearly driven by the NbO_6_ octahedrons and the A‐site cations, while in Figure 2b, the A‐site cations in K_1 − *x*_Rb_*x*_NbO_3_ and K_1 − *x*_Cs_*x*_NbO_3_ alloys do not contribute to the total *P*_s_. For the latter systems, Nb off‐centering distortions in the NbO_6_ octahedrons are the main mechanism for ferroelectricity in both K_1 − *x*_Rb_*x*_NbO_3_ and K_1 − *x*_Cs_*x*_NbO_3_ alloys.

More specifically, the above finding can be explained by considering the relatively smaller cationic size of Li (1.25 Å) and Na (1.39 Å) as compared to that of K (1.64 Å).^[^ ^41^, ^42^, ^43^ ^]^ This is nicely reflected in the study^[^ ^41^ ^]^ of Bilc and Singh where high concentration of Li was substituted in KNO (rather similar to our case). Observing the tilt instabilities due to A‐site disorder in the solid solutions, they proposed that a strong A‐site driven *P*_s_ boosting can be rationalized by the polar distortion of Li (which is in an agreement with our atomic displacement analysis presented in Figure S4, Supporting Information).^[^ ^13^, ^41^, ^42^ ^]^

As for the larger Rb (1.72 Å) and Cs (1.88 Å) cations, a larger A‐site occupancy allows for the Nb–O bond in the NbO_6_ octahedrons to increase/distort more with an overall larger volume expansion (see Figure 1c). This leads to further Nb off‐centering distortions via a second‐order Jahn–Teller effect^[^ ^36^ ^]^ as identified by the recently proposed anisotropic bond elongation index (Figure S5, Supporting Information).^[^ ^40^ ^]^ To sum things up, steric effects (from the varying sizes of cations at the A‐site) can be used to enhance polar distortions in both the A‐ and B‐site cations. These results offer an additional engineering rule of tuning the A‐site occupancy via alloying in addition to the known intra‐octahedral distortions in perovskites.

Besides these binary K_1 − *x*_A_*x*_NbO_3_ alloys, more complex ternary alloys have been investigated for their further enhanced *P*_s_ and piezoelectric performance, for example, for (K,Li,Na)NbO_3_ in contrast to (K,Na)NbO_3_.^[^ ^13^, ^14^ ^]^ (K,Na,Rb)NbO_3_ and (K,Na,Cs)NbO_3_ ternary alloys have also been explored for their improved single crystal growth via different co‐dopant ionic radii compensation mechanism.^[^ ^15^, ^16^ ^]^ If one were to go even further to consider “high entropy ceramic alloys” of KNO, it has been postulated that the multicomponent solubility limit in these complex multicomponent alloys may be improved due to the multicomponent entropy stabilizing effect.^[^ ^18^ ^]^

### 2.3 Feature‐Assisted Machine Learning Workflow

#### 2.3.1 SISSO Method

From the view point of experiments and computations, the precise engineering and atom‐by‐atom design of these complex multicomponent polar ceramics are still very challenging and demanding despite the high interest in employing them for targeted digital and information technologies. This is often impeded by synthesis challenges and the inadequacies of conventional first‐principles models. To mitigate this problem, we extend our study of binary KNO alloys by considering modern machine learning (ML) models (namely, the use of feature engineering^[^ ^44^ ^]^ via the SISSO method^[^ ^24^ ^]^) with our calculated DFPT results as inputs to examine the possibility of high ferroelectric response in multicomponent KNO alloys.

Being one of the state‐of‐the‐art ML approaches for new materials design, SISSO allows one to extract physically insightful features of a target property. It distinguishes itself from other ML methods in providing interpretable and explainable results, and thus overcoming the “impenetrable black box” of generic conventional ML models.^[^ ^24^, ^45^ ^]^ Furthermore, SISSO allows one to extract key features for even relatively sparse data^[^ ^25^, ^26^ ^]^ unlike in the case of classical ML approaches where big‐data is often needed.^[^ ^20^, ^21^, ^22^, ^23^ ^]^ Therefore, with this cutting‐edge sparse data‐driven ML technique, we proceed to perform feature extraction via the SISSO method for polar property prediction in multicomponent KNO alloys.

#### 2.3.2 Targeted Property of Interest: $\hat{P}$

**Figure 3:**
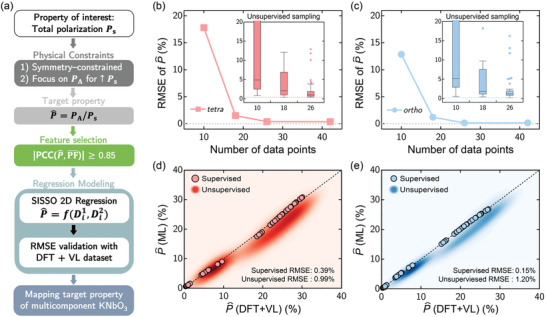
a) Feature‐assisted SISSO machine learning workflow to predict the A‐site polarization boosting, $\hat{P}$ for multicomponent KNO‐based alloys. b) For the tetragonal phase, the root‐mean‐square error (RMSE) of $\hat{P}$ (in %) is plotted as a function of the training data set size, *N*_*t*_ using the supervised data sampling method. The insert presents the RMSE values as a box‐plot where the standard deviation is applied for the whiskers and the quartiles are determined via the Tukey method. Outlier points are indicated by diamond markers. c) Similar data is shown for the orthorhombic phase, as is outlined in (b). d) For the tetragonal phase, the comparison between the predicted $\hat{P}$(ML) via the SISSO model and the calculated $\hat{P}$(DFT+VL). The data here is presented for both the supervised (with markers) and unsupervised (as a density contour map) sampling methods. The corresponding RMSE values are also listed. e) Similar data is shown for the orthorhombic phase, as is outlined in (d). Note that the reported RMSEs in (b–d) are based on the 2004 test data points generated via a Vegard's law‐like interpolation. More details can be found in the main text.

In our ML workflow in **Figure** **3**a, we start by defining the total spontaneous polarization, *P*_s_ of the KNO‐based alloys as the main property of interest. To streamline our process and make it more efficient, we apply two physical constraints to confine our study to focus on more insightful features:

1) We assume that the KNO lattice parameters of the tetragonal or orthorhombic polymorphic phases are preserved with a relatively small dopant concentration (in the A‐site), based on the multicomponent entropy stabilized effect,^[^ ^18^ ^]^
2) We consider the term $\hat{P}$, that is, the ratio of the projected spontaneous polarization due to the A‐site cations to the total spontaneous polarization of the alloy. Thus, $\hat{P}$ can be appreciated as an A‐site boosted polarization factor which can be expressed as a percentage of the *P*_s_.

As evident from Figure 2, although the contribution of NbO_6_ octahedrons to *P*_s_ is dominant and well‐understood (i.e., via intra‐octahedral tilting mechanism),^[^ ^39^, ^40^, ^46^ ^]^ the preferred use of $\hat{P}$ is palpable for the enhanced *P*_s_ in for example, K_1 − *x*_Li_*x*_NbO_3_ alloys.

#### 2.3.3 Primary Features and Pearson Correlations Coefficients

To avoid the unwanted bias on the choice of site‐averaged primary feature classes ($\bar{\mathrm{PF}}$s),^[^ ^24^, ^44^ ^]^ we proceed to filter the $\bar{\mathrm{PF}}$s using the Pearson correlations coefficients (PCCs) under the feature selection step of our workflow (Figure 3a). The calculated PCCs of $\bar{\mathrm{PF}}$s against $\hat{P}$ are tabulated in Table S4, Supporting Information and presented in Figure S6, Supporting Information. To prevent a computationally intractable feature space, we selectively omit less correlated $\bar{\mathrm{PF}}$s to $\hat{P}$ by considering a cutoff of |PCC| ⩾ 0.85, that is, $\bar{\mathrm{PF}}$s with |PCC| < 0.85 are excluded.

Based on this selection, the following $\bar{\mathrm{PF}}$s are chosen for this study: The electron affinity (*EA*, with PCC = 0.95), the Pauling electronegativity (χ, with PCC = 0.88), and the atomic radius (*r*, with PCC =−0.88). In the construction of the initial feature space, the chosen $\bar{\mathrm{PF}}$s (EA, χ, and *r*) will then be extended to each site of the perovskite structure, that is, ${\bar{\mathrm{PF}}}_{A}$, PF_Nb_, and PF_O_ to train the SISSO model.^[^ ^24^, ^45^ ^]^

Considering the influence of the A‐site on $\hat{P}$, we rationalize the chemical origin of the strongly correlated features – ${\bar{EA}}_{A}$ and ${\bar{\chi }}_{A}$ – by examining the chemical bonding characteristics between the A‐site cations and the neighboring O anions. The electron affinity, *EA* is defined as the energy released when an electron is added to a neutral atom while the Pauling electronegativity, χ of an atom is the power to attract the shared electrons within a chemical bond.^[^ ^47^ ^]^

For the lighter Group 1 elements, the A−O bonds (i.e., for Li–O and Na–O) formed will have a more covalent nature due to their higher *EA* values when compared to that of K, facilitating a back donation of electrons from O to the A‐site cations.^[^ ^48^ ^]^ In addition, their lower χ differences (i.e., χ_O_ − χ_Li_ = 2.46 and χ_O_ − χ_Na_ = 2.51) when compared to that for the K−O bond (χ_O_ − χ_K_ = 2.62) lend support to the more dominant covalent bonding character in Li−O and Na−O. These chemical bonding characteristics are also reflected in the strong negative correlation between the primary feature ${\bar{r}}_{A}$ and $\hat{P}$.

#### 2.3.4 Training Data Sets: Supervised versus Unsupervised Sampling Methods

To perform the SISSO feature extraction for the binary K_1 − *x*_A_*x*_NbO_3_ alloys, data from both the tetragonal and orthorhombic phases are used together to minimize phase‐dependency, in contrast to the actual regression where the phase‐dependent training sets are then used to evaluate the performance of the descriptor sets. In particular, for the SISSO feature extraction, we have used the 42 DFT data points, while during the validation process (via linear regression based on Equation (14)), an additional 2004 interpolated data points per phase (by assuming an almost linear behavior – Vegard‐like (VL) relation – between the DFT data points in Figure 2 have been included to validate the SISSO model's performance. More detailed information on these SISSO models and processes can be found in the Supporting Information.

In Figure 3b,c, we demonstrate the convergence of the RMSE for the SISSO regression as a function of the training data size, *N*_*t*_, for the tetragonal and orthorhombic phase, respectively. Using the 42 DFT calculated data points presented in Figure 2, we propose two ways to build the training data for convergence tests: 1) the supervised sampling method where a subset of the 42 DFT data points are systematically chosen while considering the linearity in the polarization trends (where details are outlined in the Supporting Information); and 2) the unsupervised sampling method to mimic statistical randomness where 100 unique combinations of this subset are generated for each *N*_*t*_.

Using this approach to rationalize our data sampling strategy, we clearly show that the convergence in the RMSE of the $\hat{P}$ is obtained for *N*_*t*_ used in this study. For *N*_*t*_ ⩽ 26 for each phase, it is apparent that both the supervised and unsupervised sampling method yield a high RMSE value. However, when using the supervised sampling method for *N*_*t*_ ⩾ 26, very small and converged RMSE values of 0.39% and 0.15% are achieved for both the tetragonal and orthorhombic K_1 − *x*_A_*x*_NbO_3_ alloys, respectively. Higher corresponding median (Q2) RMSE values of 0.99% and 1.20% for the tetragonal and orthorhombic phases are found for the unsupervised random sampling method.

From the inserts of Figure 3b,c, and **Table** **1**, the standard deviation (σ) and the quartile information (from the box plots) underscore the highly dispersed RMSE values for the unsupervised sampling method. We caution that, although the unsupervised sampling method may eventually yield a low RMSE value with increasing *N*_*t*_ (close to that obtained by the supervised data sampling approach), this means that the RMSE value of the unsupervised sampling method may not be an appropriate representative value given the large variances observed. It is also worthy to note that the very high σ values observed for the unsupervised sampling method in Table 1 originates from its highly skewed distribution with a very long tail.

**Table 1:** Evaluation of SISSO descriptors as a function of the training data set size, *N*_*t*_ using both the supervised and unsupervised data sampling methods for both the tetragonal and orthorhombic phases. The respective RMSE values (for the predicted $\hat{P}$ in %) are calculated by fitting the data to determine *a*_*j*_ and *b*_*j*_ in Equation (14). For the unsupervised data sampling method, the first quartile (Q1; for 25% of the data), the second quartile (Q2, also known as the median; for 50% of the data), the third quartile (Q3; for 75% of the data), and the RMSE's standard deviation (σ) are tabulated. In passing, we note that the test data set used here to assess the RMSEs is generated via a Vegard's law‐like interpolation. This is in contrast to the traditional machine learning approach where part of the training data set is normally withheld for testing

Phase	*N*_*t*_	Supervised	Q1	Unsupervised RMSE [%]		σ
		RMSE [%]		Q2	Q3	
	10	17.80	2.48	4.89	37.54	2.03 × 10^14^
Tetra	18	1.50	0.96	2.10	6.94	3.19 × 10^12^
	26	0.39	0.61	0.99	1.92	4.94 × 10^12^
	10	12.86	2.89	5.17	36.21	1.98 × 10^14^
Ortho	18	1.22	1.12	1.83	7.09	7.76 × 10^12^
	26	0.15	0.78	1.20	1.99	3.26 × 10^12^

Upon a closer inspection of the SISSO‐derived RMSE and feature ranks for different *N*_*t*_ values via the supervised training data sampling approach, we find that not only are the RMSE values well converged, the first ranked SISSO descriptors are exactly the same for both *N*_*t*_ = 26 and *N*_*t*_ = 42. This lends support to the fact that the RMSE difference between *N*_*t*_ = 26 and *N*_*t*_ = 42 is only numerical while the essential physical insight from the SISSO model is already captured for *N*_*t*_ = 26. This underscores an important fact that the SISSO method (which is based on sparse‐data compressed‐sensing formulation) has successfully “reproduced a high‐quality reconstructed signal starting from a very small set of observations”,^[^ ^26^ ^]^ highlighting that for this study, *N*_*t*_ = 26 is sufficient to train the SISSO model. Having said that, we emphasize here that the test set for the binary alloys is generated from the linear Vegard‐like (VL) relation and thus the observed linear correlation is inevitable (but physical). Hence, the observed RMSE for the binary alloy systems is considerably lower and the performance of the SISSO models here can be taken as an overestimation. However, to provide a better performance metric for these SISSO models, we will report the RMSE for the multicomponent alloy systems later in this work.

#### 2.3.5 Optimized SISSO Model: Descriptors and Prediction

Considering the first ranked SISSO regression model (*R*_1_), the *R*_1_ SISSO descriptors ($D_{1}^{1}$ and $D_{1}^{2}$) for *N*_*t*_ = 26 (and *N*_*t*_ = 42) are:

(1) $$D_{1}^{1}={\bar{\chi }}_{A}\times {\left(\frac{r_{\mathrm{Nb}}}{{\bar{EA}}_{A}}-\frac{{\bar{r}}_{A}}{EA_{O}}\right)}^{-1}\;$$

(2) $$D_{1}^{2}=\left|\left(r_{\mathrm{Nb}}+{\bar{r}}_{A}\right)\times \;\left(EA_{\mathrm{Nb}}+{\bar{\chi }}_{A}\right)-\left({\bar{r}}_{A}\times \;EA_{A}\right)\times \left(\chi_{\mathrm{Nb}}+{\bar{\chi }}_{A}\right)\right|\;$$

Using Equation (14), the $\hat{P}$ for the tetragonal and orthorhombic K_1 − *x*_A_*x*_NbO_3_ alloys can be predicted using:

(3) $${\hat{P}}_{\mathrm{tetra}}=3.85D_{1}^{1}-0.66D_{1}^{2}\;$$

and

(4) $${\hat{P}}_{\mathrm{ortho}}=3.35D_{1}^{1}-0.55D_{1}^{2}\;$$

respectively.

Thus, using Equations (3) and (4), we derive and plot the relationship between the SISSO‐predicted $\hat{P}$ and the DFT+VL‐derived $\hat{P}$ in Figure 3d,e, for the tetragonal and orthorhombic K_1 − *x*_A_*x*_NbO_3_ alloys, respectively. Most importantly, using both the supervised (as markers) and unsupervised (as density contour maps) training data sampling methods, we can clearly illustrate this agreement graphically, highlighting the advantage of the supervised sampling method over the unsupervised one. From now, with a well converged SISSO‐derived regression model using the supervised training data sets for the binary K_1 − *x*_A_*x*_NbO_3_ alloys, we will proceed to address A‐site polarization boosting ($\hat{P}$) for multicomponent KNO‐based alloys.

#### 2.3.6 Predicting A‐Site Polarization Boosting for Multicomponent Alloys

To demonstrate the use of this SISSO model to predict the $\hat{P}$ for multicomponent KNO‐based alloys, we have chosen the tetragonal penternary K_1 − *x*_(Li_*l*_Na_*m*_Rb_*n*_Cs_*o*_)_*x*_NbO_3_ system as an example. This is motivated by previous reports where less complex ternary KNO‐based alloys (e.g., (K,Na,Rb)NbO_3_ and (K,Na,Cs)NbO_3_) have been reported and proposed to be stabilized via the multicomponent entropy effect.^[^ ^15^, ^16^, ^18^, ^49^ ^]^ We anticipate that the more complex penternary K_1 − *x*_(Li_*l*_Na_*m*_Rb_*n*_Cs_*o*_)_*x*_NbO_3_ alloy will also benefit from the high entropy stabilization mechanism.

**Figure 4:**
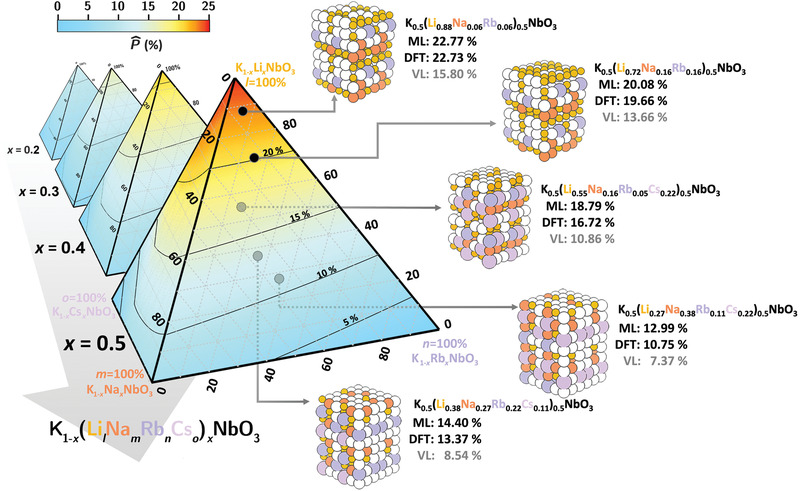
Predicted $\hat{P}$ values for the multicomponent tetragonal penternary K_1 − *x*_(Li_*l*_Na_*m*_Rb_*n*_Cs_*o*_)_*x*_NbO_3_ alloy using Equation (3) derived from the first ranked SISSO regression model (*R*_1_). Here, plots are presented as tetrahedron‐shaped quarternary $\hat{P}$ diagrams for a particular fixed *K* concentration (1 − *x*, where *x* = 0.2, 0.3, 0.4, and 0.5). SQS‐DFPT $\hat{P}$ values (and their corresponding VL‐estimated values) for five alloys are also listed to assist in the validation of the SISSO *R*_1_ predicted $\hat{P}$ values.

To predict the $\hat{P}$ via our SISSO machine learning model, we first generate a multidimensional grid of the $\bar{\mathrm{PF}}$s as a function of *x*, *l*, *m*, *n*, and *o* with the constraints of ensuring 0.2 ⩽ *x* ⩽ 0.5 and *l* + *m* + *n* + *o* = 1. Using the $\bar{\mathrm{PF}}$s for each composition, the corresponding $D_{1}^{1}$ and $D_{1}^{2}$ can be derived by using Equations (1) and (2) as inputs to Equation (3) to predict the $\hat{P}$ values for the corresponding compositions. This yields a multidimensional map of $\hat{P}$ and is plotted in **Figure** **4** as a form of a quarternary $\hat{P}$ diagram for a fixed K concentration (1 − *x*). Here, we find that, especially for Li‐rich penternary KNO‐based alloys, up to 25% A‐site polarization boosting can be achieved. A similar plot for orthorhombic K_0.5_(Li_*l*_Na_*m*_Rb_*n*_Cs_*o*_)_0.5_NbO_3_ is shown in Figure S7, Supporting Information. Similar to the binary alloys, the $\hat{P}$ values for the tetragonal alloys are somewhat higher than that of the orthorhombic ones.

To validate our SISSO model predicted $\hat{P}$ values, using the aforementioned SQS method, we have calculated the $\hat{P}$ values for three penternary (K_0.5_(Li_0.55_Na_0.16_Rb_0.05_Cs_0.22_)_0.5_NbO_3_, K_0.5_(Li_0.27_Na_0.38_Rb_0.11_Cs_0.22_)_0.5_NbO_3_, and K_0.5_(Li_0.38_Na_0.27_Rb_0.22_Cs_0.11_)_0.5_NbO_3_) and 2 quaternary (K_0.5_(Li_0.88_Na_0.06_Rb_0.06_)_0.5_NbO_3_ and K_0.5_(Li_0.72_Na_0.16_Rb_0.16_)_0.5_NbO_3_) alloys for the tetragonal and orthorhombic phases. This result in a total of ten DFPT calculations with up to 180 atoms in the supercells considered. These DFPT data are then used to validate both the $\hat{P}$ values derived from the trained SISSO model and classic Vegard's law (VL) model. The VL model is based on the linear interpolation of *P*_A_ and *P*_s_ as a function of K concentration. As listed alongside the corresponding structures used for the validation, the $\hat{P}$ values obtained via the VL model is typically underestimated (≈4.53%) when compared to the DFT‐derived values, while those predicted by the SISSO model perform very well with an error of only 1.61% as referenced to the DFT ones.

### 2.4 Extracting Physical Insights from the Machine Learning Models

By construction, SISSO is an ideal machine learning method to derive physically intuitive descriptors.^[^ ^24^ ^]^ The descriptors of a SISSO regression model are, thus, in principle physically intuitive. However, when it is used for linear fitting, the descriptors are multiplied by the coefficients (cf. Equations (3) and (4), for instance), masking the direct physical interpretation of the descriptors (cf. Equations (1) and (2), for example). In contrast, the descriptors of a SISSO classification model are used directly to set the boundaries and thus mitigating this loss of interpretation. Within the constraints of linearity, the SISSO regression model with a small data set may achieve a higher level of accuracy in prediction while the SISSO classification model which depends intricately on the dividing boundaries will generally require a larger data set to train.^[^ ^50^, ^51^ ^]^

**Figure 5:**
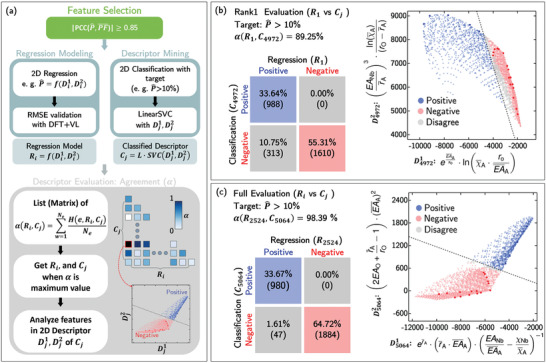
a) Revised feature‐assisted SISSO machine learning workflow for $\hat{P}$ predictions that include the calculation of the agreement index, α(*R*_*i*_, *C*_*j*_) where the level of agreement between the SISSO regression model (*R*_*i*_) and the SISSO classification model (*C*_*j*_) is examined via the agreement matrix. b) The agreement matrix for the (*R*_1_, *C*_4972_) pair is shown for $\hat{P}>10\%$. The SISSO classification plot for *C*_4972_ using Equations (7) and (8) is also presented. c) Similarly, for the highest value of $\alpha (R_{2524},\;C_{5064})=99.86\;\%$, the agreement matrix for the (*R*_2524_, *C*_5064_) pair is depicted for $\hat{P}>10\%$. Likewise, the SISSO classification plot for *C*_5064_ using Equations (9) and (10) is also plotted.

In this study, by setting a lower boundary for $\hat{P}$, that is, $\hat{P}>10\;\%$, the SISSO model generates 6 336 486 797 different possible descriptors for this condition. To ensure that our SISSO model remains tractable, we have limited our study to the top 10 000 descriptors. These are then assessed by a linear support vector classification (LSVC) machine algorithm. Due to a clear separation in the data points and high linearity, classification and regression models are all converged and perform well. Thus, we need to develop a method to not only allow us to make accurate predictions using the regression model but to also present a physically interpretable classification model. The updated machine learning workflow is now presented in **Figure** **5**a.

To do this, we first introduce a new concept of agreement as represented by the agreement index, α(*R*_*i*_, *C*_*j*_) where we compare the level of agreement between the regression model (*R*_*i*_) and classification model (*C*_*j*_) via the agreement matrix. This agreement matrix resembles the well‐known confusion matrix that has been widely used to assess classification models. Mathematically,

(5) $$\alpha \left(R_{i},\;C_{j}\right)=\sum_{e=1}^{N_{e}}\frac{H(e,\;i,\;j)}{N_{e}}\;$$

where *e* represents a unique alloy composition point including the associated primary features, $\left\{{\bar{EA}}_{A},\;{\bar{\chi }}_{A},\;{\bar{r}}_{A},\;\text{…}\right\}$, in an evaluation data set of K_1 − *x*_(Li_*l*_Na_*m*_Rb_*n*_Cs_*o*_)_*x*_NbO_3_ polarization diagram (in Figure 4), *N*_e_ is the total number of evaluation points, and *H*(*e*, *i*, *j*) is a pair parameter to judge the agreement between each pair of regression model (*R*_*i*_) and classification model (*C*_*j*_). Here, *H*(*e*, *i*, *j*) could be expressed as:

(6) $$H\left(e,\;i,\;j\right)=\left\{\begin{array}{cc} 1 & \mathrm{if}\;\;\left(R_{i}\left(e\right)-{\hat{P}}_{t}\right)C_{j}\left(e\right)\geq 0\\ 0 & \mathrm{if}\;\;\left(R_{i}\left(e\right)-{\hat{P}}_{t}\right)C_{j}\left(e\right)<0 \end{array}\right.\;$$

where *R*_*i*_(*e*) represent the predicted $\hat{P}$ from the given regression model (*R*_*i*_) for the *e* point, ${\hat{P}}_{t}$ is the desired $\hat{P}$ boundary value (e.g., 10%), and *C*_*j*_(*e*) represents the classification result from the classification model (*C*_*j*_) for the *e* point, taking +1 when true and −1 when false. Using this definition of *H*(*x*, *i*, *j*) (in Figure 5a) in combination with the 2911 points of K_1 − *x*_(Li_*l*_Na_*m*_Rb_*n*_Cs_*o*_)_*x*_NbO_3_, we can now calculate α(*R*_*i*_, *C*_*j*_) (cf. Equation (5)) and determine the agreement matrix for a given condition (i.e., $\hat{P}>10\;\%$) in Figure 5b,c.

To illustrate this concept of agreement, in Figure 5b, we first determine the 1st ranked regression model, *R*_1_ and calculate α(*R*_1_, *C*_*j*_) iteratively for 10 000 generated *C*_*j*_ under the condition that $\hat{P}>10\%$. The highest $\alpha (R_{1},\;C_{j})=89.25\%$ is found for the (*R*_1_, *C*_4972_) pair and the agreement matrix is plotted in the left bottom corner of Figure 5b. Here, the *C*_4972_ provides two descriptors:

(7) $$D_{4972}^{1}:\exp\left(\frac{{\bar{EA}}_{A}}{\chi_{O}}\right)\times \ln\left({\bar{\chi }}_{A}\times \frac{r_{O}}{{\bar{EA}}_{A}}\right)\;\;\mathrm{and}$$

(8) $$D_{4972}^{2}:{\left(\frac{EA_{\mathrm{Nb}}}{{\bar{r}}_{A}}\right)}^{3}\times \;\frac{\ln\left({\bar{\chi }}_{A}\right)}{\left(r_{O}-{\bar{r}}_{A}\right)}\;$$

To extract the physical insights from this SISSO classification model, we take a closer look at Equation (7). Here, in order for $D_{4972}^{1}$ to shift to more negative values (i.e., moving from the right (red) to the left (blue) region), ${\bar{EA}}_{A}$ in the $\exp(\frac{{\bar{EA}}_{A}}{\chi_{O}})$ term will have to take on larger values, since $\ln\left({\bar{\chi }}_{A}\times \;\frac{r_{O}}{{\bar{EA}}_{A}}\right)$ is always negative. Thus, a more negative $D_{4972}^{1}$ with higher ${\bar{EA}}_{A}$ values will predict a higher $\hat{P}$.

Turning our attention to Equation (8), we notice that a small ${\bar{r}}_{A}$ value will shift $D_{4972}^{2}$ to larger values due to the cubic ${\left(\frac{EA_{\mathrm{Nb}}}{{\bar{r}}_{A}}\right)}^{3}$ term, noting that the $\frac{\ln({\bar{\chi }}_{A})}{\left(r_{O}-{\bar{r}}_{A}\right)}$ term is always positive. Thus, a smaller ${\bar{r}}_{A}$ will suggest a higher $\hat{P}$ value, aligning with our Pearson correlation analysis where $\hat{P}$ and ${\bar{r}}_{A}$ are highly correlated (see Table S4, Supporting Information).

Given the determined α(*R*_1_, *C*_4972_) is not the highest value for our data set, we remove the constraint of simply replying on *R*_1_ and proceed to calculate the α(*R*_*i*_, *C*_*j*_) values for all possible (*R*_*i*_, *C*_*j*_) pair combinations of 10 000 regression models and 10 000 classification models to uncover the highest possible value of α. After a rigorous assessment of 10^8^ unique combinations of (*R*_1_, *C*_*j*_) pairs, the highest agreement index value of 99.86 % is found for the (*R*_2524_, *C*_5064_) pair under the condition that $\hat{P}>10\;\%$. The agreement matrix and the SISSO classification plot are presented in Figure 5c.

Having a very high α(*R*_2524_, *C*_5064_) value of 99.86 %, we now turn our attention to the descriptors of *C*_5064_:

(9) $$D_{5064}^{1}:\exp\left({\bar{r}}_{A}\right)\times \left({\bar{r}}_{A}\times \;{\bar{EA}}_{A}\right)\times \;{\left(\frac{EA_{\mathrm{Nb}}}{{\bar{EA}}_{A}}-\frac{\chi_{\mathrm{Nb}}}{{\bar{\chi }}_{A}}\right)}^{-1}\;\;\mathrm{and}$$

(10) $$D_{5064}^{2}:\left(2EA_{O}+\frac{{\bar{r}}_{A}}{r_{O}}-1\right)\times \;{\left({\bar{EA}}_{A}\right)}^{2}$$

We note that the corresponding *R*_2524_ equations are provided in the Supporting Information. Upon a closer inspection of Equation (9), given that the term $\frac{EA_{\mathrm{Nb}}}{{\bar{EA}}_{A}}-\frac{\chi_{\mathrm{Nb}}}{{\bar{\chi }}_{A}}$ is always negative and the term $\exp\left({\bar{r}}_{A}\right)\times \left({\bar{r}}_{A}\times {\bar{EA}}_{A}\right)$ will also always take on a positive value, smaller values of ${\bar{r}}_{A}$ in the exponential term $\exp({\bar{r}}_{A})$ will shift $D_{5064}^{1}$ from the left (red) to the right (blue) region. This provides a feature‐assisted prediction of a higher $\hat{P}$ value from a smaller ${\bar{r}}_{A}$ indicating a higher likelihood of A‐site boosted polarization. This nicely corroborates with the Pearson correlation analysis where ${\bar{r}}_{A}$ is one of the key primary features that is highly correlated with $\hat{P}$.

On the other hand, in Equation (10), an increase in $D_{5064}^{2}$ (i.e., shifting from the bottom (red) to the upper (blue) region) is a consequence of a corresponding increase in the term ${\left({\bar{EA}}_{A}\right)}^{2}$ where a quadratic dependence is established. Similarly, a higher $D_{5064}^{2}$ will predict a larger $\hat{P}$ value, again lending support to the established high Pearson correlation coefficient between $\hat{P}$ and ${\bar{EA}}_{A}$.

In essence, by selecting the representative (*R*_*i*_, *C*_*j*_) pair, SISSO provides a consistent approach to suggest physically insightful descriptors (aligning with the Pearson correlation analysis). High A‐site boosted polarization (i.e., high $\hat{P}$ values) in multicomponent KNO‐based alloys can be designed by choosing A‐site cation dopants with small ${\bar{r}}_{A}$ and large ${\bar{EA}}_{A}$. In the same vein, it is noticed that higher values of ${\bar{\chi }}_{A}$ will also lead to high A‐site boosted polarization.

## 3 Perspective: Toward Higher Polarity in Lead‐Free Oxides for Ferroelectric Applications

In this work, we have discussed the training of a feature‐assisted SISSO model for multicomponent KNO‐based alloys in hope to achieve high A‐site polarization boosting via the target property, $\hat{P}$. We have shown that through the use of the supervised data sampling method and by applying the concept of agreement, we have enabled our predictive SISSO model to determine the $\hat{P}$ of multicomponent KNO‐based alloys with higher level of statistical confidence for a physically interpretable ML model.

**Figure 6:**
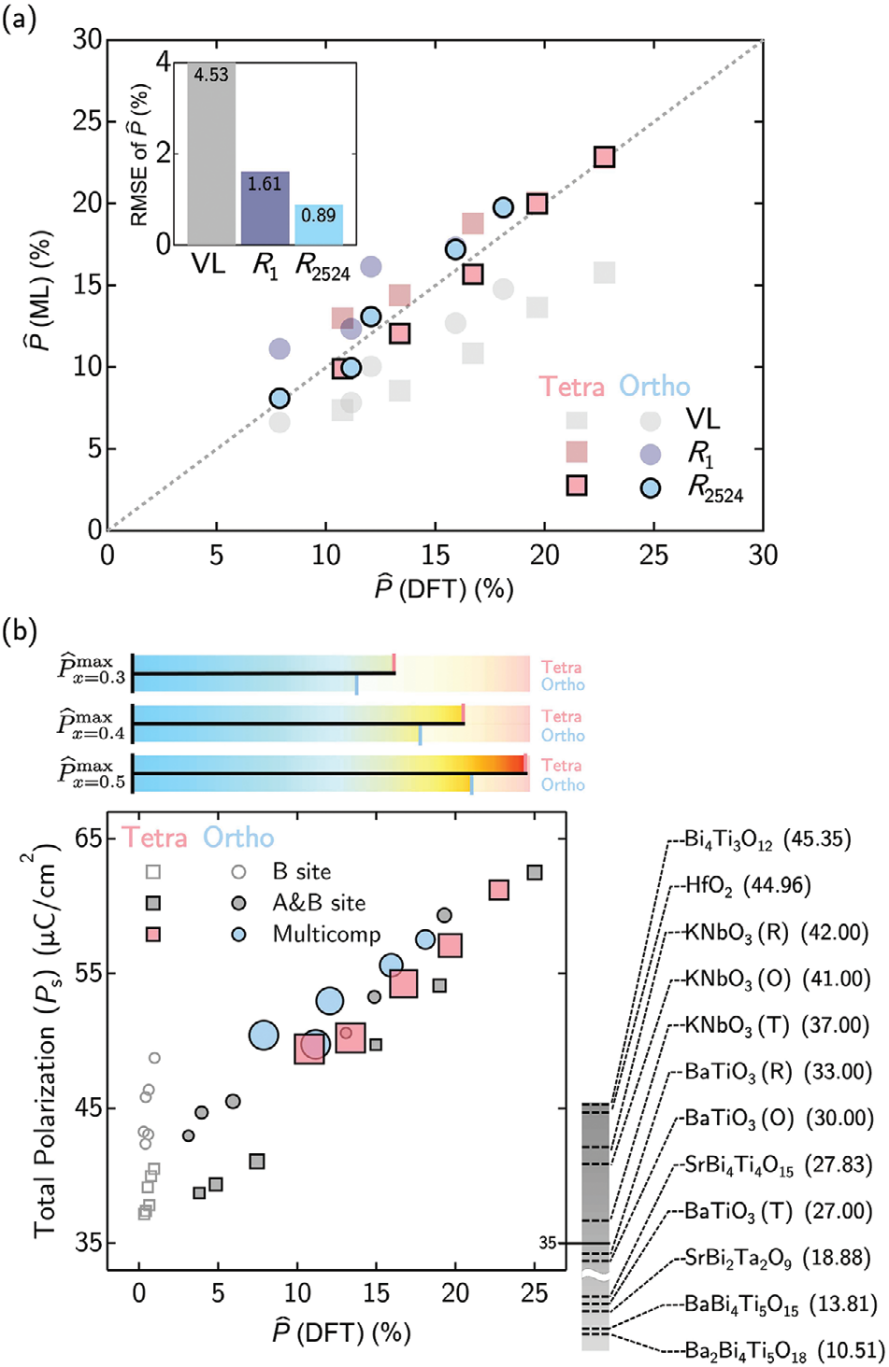
a) Comparison between the DFPT‐calculated $\hat{P}$ values of multicomponent KNO‐based alloys versus the model‐predicted $\hat{P}$ values using the conventional Vegard's relation (VL), the first ranked SISSO regression model (*R*_1_), and the SISSO regression model *R*_2524_ which yielded the highest agreement index value. The insert shows the RMSE values of $\hat{P}$ as predicted by these models. b) Plot of the DFPT‐calculated total spontaneous polarization (*P*_s_) versus the A‐site polarization boosting ($\hat{P}$) for the various binary and multicomponent KNO‐based alloys for both the tetragonal and orthorhombic phases to illustrate the potential enhancement of ferroelectric properties in these oxide alloys. The markers are weighted according to the ideal mixing entropy at *T* = 300 K where larger markers are deemed to be more entropy stabilized. To afford a comparison with the current well‐known lead‐free ferroelectrics from literature, their experimental *P*_s_ are also shown alongside the plot (The *P*_s_ values in μC cm^−2^ are taken from refs. [52, 53]). The colormaps used for the data markers follow that in Figure 4.

Through this work, we have also challenged the conventional Vegard's relation (VL) which is commonly used for determining various properties of alloys and solid solutions. In **Figure** **6**a, it is clear that when the $\hat{P}$ determined via the VL model alone as compared to the actual DFPT calculated values, an underestimation of $\hat{P}$ (with a RMSE of 4.53%) is found. The SISSO‐based *R*_1_ and *R*_2524_ provides a much closer agreement to the DFPT values, with a RMSE of 1.61% and 0.89%, respectively. The further improvement found for *R*_2524_ stems from the higher agreement index where an analytical comparison between SISSO regression and SISSO classification models offers a higher accuracy via the regression model and a physically interpretable classification model at the same footing.

So far, we have focused only on the reduced target property indicator – the A‐site polarization boosting factor, $\hat{P}$. However, given that the dominant contribution to the total spontaneous polarization, *P*_s_ comes mainly from the NbO_6_ octahedral distortions (as seen in Figure 2), a high $\hat{P}$ may not guarantee a high value for *P*_s_ which is a key ingredient for ferroelectric applications. Thus, using DFPT calculations, we now present both the *P*_s_ and the corresponding $\hat{P}$ for both binary, quarternary, and penternary KNO‐based alloys in both the tetragonal and orthorhombic phases, and the ideal entropy (at *T* = 300 K) stabilization‐weighted scatter plot is graphed in Figure 6b.

The calculated total spontaneous polarization, *P*_s_ is found to vary almost linearly with the A‐site polarization boosting factor, $\hat{P}$. And interestingly, multicomponent KNO‐based alloys can indeed afford a strong enhancement to the overall spontaneous polarization value, for example, by up to 150% (referenced to pristine KNbO_3_) for $\hat{P}≃25\%$ in K_0.5_(Li_0.88_Na_0.06_Rb_0.06_)_0.5_NbO_3_. Besides a promising enhancement in *P*_s_, it is also worth mentioning that the multicomponent KNO‐based alloys exhibit a general tendency for higher stability via the high entropy stabilizing effect.^[^ ^15^, ^16^, ^18^ ^]^ For instance, in the case of tetragonal quarternary K_0.5_Li_0.44_Na_0.03_Rb_0.03_NbO_3_ and binary K_0.5_Li_0.5_NbO_3_, we find that both alloys have relatively high *P*_s_ values of 61.21 and 62.52 μC cm^−^2, respectively. However, the quarternary alloy is determined to have a 30% higher mixing entropy value than the binary alloy which leads to the conjecture that the quarternary alloy will be more entropy‐stabilized.

To provide a perspective on how these multicomponent K_1 − *x*_(Li_*l*_Na_*m*_Rb_*n*_Cs_*o*_)_*x*_NbO_3_ alloys fare as compared to experimentally reported lead‐free ferroelectrics,^[^ ^52^, ^53^ ^]^ we have listed their experimentally determined *P*_s_ alongside our scatter plot in Figure 6b for comparison (detailed values are provided in Table S6, Supporting Information). Indeed, it is very clear that the lead‐free multicomponent KNO‐based alloys proposed in this work are very promising and may be a very strong contender to outperform the currently known ones from experiments.

## 4 Conclusions

Through the use of ab initio density‐functional perturbation theory calculations and physically interpretable feature‐assisted machine learning models, we systematically examine and investigate the origins of A‐site enhanced polarization mechanism in multicomponent KNbO_3_‐derived K_1 − *x*_(Li_*l*_Na_*m*_Rb_*n*_Cs_*o*_)_*x*_NbO_3_ alloys. Starting from the simpler analogs of binary K_1 − *x*_A_*x*_NbO_3_ (where A = Li, Na, Rb, and Cs) generated by SQS method, we determine that they are entropy‐stabilized and exhibit large values of spontaneous polarization (comparable to or higher than that of BaTiO_3_). Using the SISSO method to extract physically meaningful and interpretable descriptors based on primary elemental features for predicting the polarization enhancement due to the A‐site cation, $\hat{P}$, we demonstrate numerical convergence for our data set size and provide a statistical analysis via both supervised and unsupervised data sampling methods, achieving a low RMSE value of less than 1.61%. Using the SISSO‐determined descriptors for the binary alloys, we have naturally extended this to multicomponent alloys and have provided a multidimensional $\hat{P}$ prediction diagram. We cross‐validate our SISSO predictions using both the conventional Vegard's relation and ab initio DFPT values. Using a new metric of agreement via both SISSO regression and classification models for $\hat{P}>10a$%, we have further narrowed the prediction RMSE to 0.89%. Importantly, through this feature‐driven machine learning scheme, we have incontrovertibly demonstrated that precise engineering of the A‐site cation composition in KNbO_3_ can result in a very high boosting to the total spontaneous polarization values (more than 150% when compared to pristine KNbO_3_) and these lead‐free multicomponent K_1 − *x*_(Li_*l*_Na_*m*_Rb_*n*_Cs_*o*_)_*x*_NbO_3_ alloys are truly potential contenders for the currently known lead‐free counterparts for the next‐generation ferroelectric applications such as ferroelectric random access memory and piezoelectric energy converters.

## 5 Experimental Section

### Density‐Functional Theory Calculations

Density‐functional theory (DFT) calculations were performed using periodic boundary conditions, employing the projector augmented wave (PAW)^[^ ^54^, ^55^ ^]^ method as implemented in the Vienna Ab initio Simulation Package code.^[^ ^56^, ^57^ ^]^ The Kohn–Sham orbitals were expanded using a plane‐wave basis set with a kinetic energy cutoff of 700 eV. The 1s, 2s, and 2p states of Li, the 2p and 3s states of Na, the 3s, 3p, and 4s states of K, the 4s, 4p, 5s states of Rb, the 5s, 5p, 6s states of Cs, the 4p, 4d, and 5s states of Nb, and the 2s and 2p states of O were explicitly considered as valence states within the PAW approach.

For the DFT exchange‐correlation (*xc*) functional, it had been reported in a previous work^[^ ^40^ ^]^ that the Perdew–Burke–Ernzerhof *xc* functional revised for solids (PBEsol)^[^ ^58^ ^]^ provided the best agreement with the experimental lattice parameters of KNbO_3_ polymorphs. Brillouin zone integrations were sampled with a Γ‐centered **k**‐point mesh of 8 × 8 × 8 for the primitive unit cell of tetragonal and orthorhombic phases, and for larger supercells used in this work, the **k**‐point meshes were then equivalently folded.

### Structure Modeling for Solid Solutions

To account for the structural models of solid solutions, K_1 − *x*_A_*x*_NbO_3_ (where A = Li, Na, Rb, and Cs), the special quasi‐random structures (SQSs)^[^ ^59^, ^60^ ^]^ method was used. SQSs were periodic structures with selected atomic distributions from the cluster correlations approach where the randomized atomic arrangement mimics the most disordered structure among all inequivalent configurations for a given composition. The SQS method is widely used for solid solution or alloy formation modeling.^[^ ^61^, ^62^, ^63^ ^]^ Based on the parent structures of the primitive KNbO_3_ tetrahedral and orthorhombic polymorphs, the integrated cluster expansion toolkit (ICET) code^[^ ^64^ ^]^ was employed to search for the optimal supercell that best depicts this random structure.

The initial lattice constants of the SQSs were approximated by a weighted average of the optimized lattice constants of the parent oxides based on Vegard's law.^[^ ^37^ ^]^ The atomic coordinates and volume of the constructed alloys were then allowed to be fully relaxed. Here, their randomness is verified by inspecting the atomic pair correlation function (APCF) of these SQSs. Further details and the corresponding supercell configurations can be found in the Supporting Information (e.g., see Table S2, Supporting Information).

### Spontaneous Polarization

The spontaneous polarization (*P*_s_) was obtained by considering the displacement of each atom (δ*d*) from the position of the ideal non‐polar centrosymmetric structure and the averaged values of BEC tensor of each atom (Z*), respectively.^[^ ^65^, ^66^ ^]^ Formally, *P*_s_ simply took the form of

(11) $$P_{i}=\sum_{i}\frac{e}{\Omega }Z_{i}^{*}\delta d_{i}\;$$

where *i* denotes the *i*th atom, $Z_{i}^{*}$ the Born effective charges derived from density‐functional perturbation theory calculations^[^ ^67^, ^68^ ^]^ associated with the *i*th atomic displacement (δ*d*_*i*_) of the ions from their position in the unpolarized (non‐polar) structure, *e* the charge of an electron, and Ω the cell volume considered. By comparing to the non‐polar reference structure, the atomic displacements responsible for ferroelectricity could be thus analyzed.

### Compressed‐Sensing Machine Learning: Descriptors and Features

Material characteristics and attributes (or more commonly termed as features) play an important role in determining the accuracy of a descriptor‐based machine learning model.^[^ ^22^, ^24^, ^44^ ^]^ In general, the elemental, structural, electronic, or other features of materials could be considered. In this work, the focus was only on the primary (or elemental/atomic) features that allow to predict the target property of interest here, that is A‐site element contribution to the total polarization, $\hat{P}$. Table S5, Supporting Information, tabulates the considered primary features (PFs) used in this work.

Site‐specific descriptors of K_1 − *x*_A_*x*_NbO_3_ cation solid solution (where A = Li, Na, Rb, and Cs), descriptors for each site was transformed into a weighted average according to

(12) $${\bar{\mathrm{PF}}}_{\beta }=\sum_{i=1}^{N}\zeta_{i}{\mathrm{PF}}_{i}\;$$

where β is A, B, and X for a typical ABX_3_ perovskite. The averaged PFs for the each site specific properties and the coefficient ζ (which takes a value between 0 and 1) denote the fractional occupancy of the each site in the alloy. This was done to ensure that the compositional variance between the data was kept, and thus allowing to also map out the polarization with regards to any fractional stoichiometry based on essential physical factors.^[^ ^69^ ^]^ For this paper, ${\bar{\mathrm{PF}}}_{A}$ varied due to the A‐site substitution, while ${\bar{\mathrm{PF}}}_{B}$ and ${\bar{\mathrm{PF}}}_{X}$ were constants.

To assess the overall primary feature relations to the target feature, primary feature class ($\bar{\mathrm{PF}}$) is defined as the following:

(13) $$\bar{\mathrm{PF}}=\frac{1}{3}\left({\bar{\mathrm{PF}}}_{A}+{\bar{\mathrm{PF}}}_{B}+{\bar{\mathrm{PF}}}_{X}\right)$$

Based on the transformed descriptors ($\bar{\mathrm{PF}}$; cf. Equation (13)), Pearson correlation coefficients (PCCs) were then surveyed to choose the relevant features having a higher correlation with the target property, $\hat{P}$ (here, the top $\bar{\mathrm{PF}}$s are chosen, for |PCC| ⩾ 0.85) while removing less correlated features (i.e., the other $\bar{\mathrm{PF}}$s, where |PCC| < 0.85). This pre‐processing step made the SISSO process more effective by preventing the non‐distinctive features from increasing the dimensions of the models meaninglessly. This had shown to greatly help in the reduction of computational memory during feature generation.^[^ ^24^, ^45^ ^]^ It was important to note that the primary feature class ($\bar{\mathrm{PF}}$) itself may not retain all the physical information but only to assist in the pre‐processing step of the SISSO process.

With the chosen PFs, the SISSO generated a combination of features by applying several mathematical operators (which are +, −, ×, /, exp , exp −, ^−1^, ^2^, ^3^, √, $\sqrt[{3}]{}$, log, | − |) iteratively. To construct arbitrarily large feature spaces, three iterations (or dimensions) were considered, thereby generating the feature spaces Φ_1_, Φ_2_, and Φ_3_ (where Φ_*n*_ corresponds to the *n*th dimension feature space). Note that for a given feature space, Φ_*n*_, it will automatically include the lower dimension feature spaces. Finally, for this work, the SISSO generated 126 122 065 features and ranked them according to the root‐mean‐square error (RMSE) to find the best descriptor sets. Since a 2D regression was applied to the given training set, the final regression result is expressed as

(14) $$\hat{P}=a_{j}D_{j}^{1}+b_{j}D_{j}^{2}\;$$

where *a*_*j*_ and *b*_*j*_ are the fitted coefficients of regression using two *j*th rank descriptor sets ($D_{j}^{1}$, $D_{j}^{2}$) and the training set. To obtain the ranking of SISSO descriptors, both the data of the tetragonal and orthorhombic phases were used to minimize phase‐dependency, in contrast to the actual regression where the phase‐dependent training set was used to evaluate the performance of the descriptor sets.

### Statistical Analysis

The data in this manuscript can be divided into three main sections: i) DFT‐generated polarization values for 42 different binary alloys; ii) linearly‐interpolated data based on a Vegard's law‐like model (resulting in 2004 points per phase—for both the tetragonal and the orthorhombic phases, respectively. Details regarding the interpolated data can be found in the Supporting Information); iii) ten validation samples of polarization values for selected multicomponent alloys. In (i), the data were used for the training of SISSO, while in (ii), the 2004 interpolated data points were used to test the results of the SISSO for the binary alloys. In (iii), the ten DFT‐calculated polarization values for selected multicomponent alloys were used to validate the accuracy of the SISSO in part to establish its predictive nature for the multicomponent alloys.

We note that, in conventional machine learning with DFT data, the DFT data was normally split into the training and test sets. However, due to computational limitations, all DFT data for the binary alloys (in (i)] had been used for the training process and then the validity of the training results were cross checked using data from (ii) and (iii). For all of these data in (ii) and (iii), no additional pre‐processing was applied to prevent any loss of physical meaning. For the regression modeling, the python package sklearn has been used. For the SISSO calculations, the SISSO package developed by Ouyang et al.^[^ ^24^ ^]^ has been employed.

## Conflict of Interest

The authors declare no conflict of interest.

## Author Contributions

S.‐H.V.O and W.H. contributed equally to this work. S.‐H.V.O., W.H., and K.K. constructed the atomistic models and performed the calculations. A.S. and J.‐H.L. conceptualized and supervised this work. All authors were involved in the drafting of the manuscript.

## Supporting information

Supporting Information

## Data Availability

The data that support the findings of this study are openly available in zenodo at https://doi.org/10.5281/zenodo.5512153, reference number 5512153.
