# Supplementary material for: Using Feature‐Assisted Machine Learning Algorithms to Boost Polarity in Lead‐Free Multicomponent Niobate Alloys for High‐Performance Ferroelectrics
Source: Adv Sci (Weinh). 2022 Mar 6;9(13):2104569. doi: 10.1002/advs.202104569 (PMC9434731; doi:10.1002/advs.202104569)
Supplement: Supplementary file 1 — Supporting Information [file ADVS-9-2104569-s001.pdf]

Supplementary Information

Using feature-assisted machine learning algorithms to boost polarity in lead-free multicomponent niobate alloys for high-performance ferroelectrics

Seung-Hyun Victor Oh,* Woohyun Hwang,*
Kwangrae Kim, Ji-Hwan Lee,† and Aloysius Soon‡
*Department of Materials Science & Engineering
and Center for Artificial Synesthesia Materials,
Yonsei University, Seoul 03722, Republic of Korea*

S1. OPTIMIZED LATTICE PARAMETERS

TABLE S1. Optimized lattice parameters (a_0, b_0, c_0 in Å, and α, β, and γ in °), and crystallographic space group of bulk $KNbO_3$ polymorphs, calculated using PBEsol xc functional with comparison of other theoretical and experimental literatures. All theory results are taken from Reference 1. Other relevant experimental values are also listed and taken from References 2 and 3.

xc	Phase	a_0 (Å)	b_0 (Å)	c_0 (Å)	α (°)	β (°)	γ (°)
Our work	*Tetra*	3.969	3.969	4.058	90	90	90.000
	Ortho	4.022	4.022	3.961	90	90	90.163
PBEsol [1]	*Tetra*	3.969	3.969	4.058	90	90	90.000
	Ortho	4.021	4.021	3.961	90	90	90.180
Experiment [2, 3]	*Tetra*	3.997	3.997	4.063	90	90	90.000
	Ortho	4.035	4.035	3.973	90	90	90.270

* Contributed equally to this work
† Corresponding: jkwnlee88@yonsei.ac.kr
‡ Corresponding: aloysius.soon@yonsei.ac.kr

S2. SPECIAL QUASI-RANDOM STRUCTURE (SQS)

In this work, we use the special quasi-random structures (SQSs) [4, 5] method to model the solid solution of $K_{1-x}A_xNbO_3$ (A = Li, Na, Rb, Cs). SQSs method (first developed by Zunger *et al.*[4]) allows us to mimic random solutions without infinitely expanding the supercell and determine the most disordered structure among all inequivalent configurations for a given composition.

Due to our primary interest in A cation substitution, the structure was treated as a pseudo-binary system. Based on the parent structure of $KNbO_3$ tetrahedral, and orthogonal primitive cells, the integrated cluster expansion toolkit (ICET) code [6] are utilized to search for the optimal supercell that best depicts the random structure. Different super-cell configurations are considered and tested from 2×2×2 to 4×4×4. From these diverse configurations, the representative structures are chosen regarding two factors: the total number of atoms (for computational limits) and the Atomic Correlation Function.

Atomic pair correlation functions (APCF) are used to characterize the statistical arrangement of atoms. The most disordered structure by using atomic pair correlation functions [4, 5] can be found, because APCF allows us to quantify the structural degree of random ordering. From the lattice algebra each site is assigned a pseudo-spin variable of $S_i = -1/+1$ depending on which atom (A/B) is occupying the site. In addition, these sites are grouped into figures, $f(k, m)$. Where $k = 1, 2, 3, .,$ is the vertices and $m = 1, 2, 3, .,$ is the spanning maximum distances (first, second, and third-nearest neighbors, etc). The sum of the products of spin variables over all sites related to the figure of the lattice gives the correlation function $\overline{\Pi}_{k,m}$.

Basically, APCF of fully random binary alloys considering 2-atom interactions is approximated to $\overline{\prod}_{2,m} = (2x-1)^2 = \overline{\prod}_{\text{ideal}}$ where m indicates the mth shell of nearest neighbor atoms and x is the substituent composition. APCF are calculated based on Equation S1 shown below,

$$\overline{\Pi}_{2,m} = \frac{1}{ND_m} \sum_{i,j \in r_{i,j}=R_m} S_i S_j \quad , \tag{S1}$$

where N is the number of cation lattice sites, D_m is the number of pairs among atoms i and j within the mth shell, $r_{i,j}$ is the distance between atoms i and j and R_m is the radius of the mth shell of sphere. The pair parameter S_i takes the value +1 when i is an atom of type A and −1 for an atom of type B.

TABLE S2. The atom pair correlation functions (APCF) of $KNbO_3$ tetragonal ($P4$mm) and orthorhombic ($Amm2$) based solid solution ($K_{1-x}A_xNbO_3$, A = Li, Na, Rb, Cs) and their corresponding supercell configurations

Phase	Supercell	x	$\prod_{ideal}$	$\prod_{2,1}$	$\prod_{2,2}$	$\prod_{2,3}$	$\prod_{2,4}$	$\prod_{2,5}$
Tetragonal	2x2x4	0.25	0.250	0.250	0.250	0.000	0.250	0.250
	3x2x3	0.33	0.111	0.111	0.111	0.111	0.111	0.111
	4x2x2	0.5	0.000	0.000	0.000	0.000	0.000	0.000
	3x2x3	0.66	0.111	0.111	0.111	0.111	0.111	0.111
	2x2x4	0.75	0.250	0.250	0.250	0.000	0.250	0.250
Orthorhombic	2x2x4	0.25	0.250	0.250	0.250	0.250	0.000	0.000
	3x3x2	0.33	0.111	0.111	0.111	0.111	0.111	0.111
	4x2x2	0.5	0.000	0.000	0.000	0.000	0.000	0.000
	3x3x2	0.66	0.111	0.111	0.111	0.111	0.111	0.111
	2x2x4	0.75	0.250	0.250	0.250	0.250	0.000	0.000

Though it is meaningless to mention that the consideration of many-body interactions would increase the randomness of the generated structure, this would require much larger supercells resulting in an unreal computational cost. In order to limit the computational cost of these solid solutions, the numbers of atoms are restricted to 80 - 100 atoms in each supercell.

For the compositions of $x = 0.25$, 0.33, 0.50, 0.66, and 0.75 in $K_{1-x}A_xNbO_3$ (A = Li, Na, Rb, Cs), our modeled structures are chosen to match the correlations of the random alloy out to the second-nearest neighbor. Based on the considerations, $2 \times 2 \times 4$, 3×3×2 (3×2×3 for tetragonal), 4×2×2, 3×3×2 (3×2×3 for tetragonal), 2×2×4 supercells for compositions of 0.25, 0.33, 0.50, 0.66, and 0.75 are selected for all phases of tetragonal and orthorhombic.

The corresponding atomic pair correlations and their supercell configurations can be found in the Table S2. This limitation to the number of atoms may result in the artificially randomized structures only matching the ideal correlation functions up to the second-nearest

neighbor. Though this result may be considered short ranged, it is well known from the work of Zunger *et al.* [4] that interatomic electronic interactions only take place in short range. The calculated properties such as enthalpy of mixing, charge transfer, etc. depend mostly on local atomic arrangements. Thus, even with the correlation function of the generated structure matching to the second neighbor, we can assume that the structure represents a highly randomized solid solution.

Also, we note that for different A cation substitutes, modeled alloy systems were not separately generated but the representative SQSs are used. Since SQSs only consider the correlation of different types of atoms, regardless of chemical characteristics, in theory, creating a $A_{1-x}B_xNbO_3$ pseudo-binary system structure then replacing the B site with Li, Na, Rb, Cs should all represent the random structure identically.

S3. GOLDSCHMIDT AND NEW TOLERANCE FACTOR

TABLE S3. Ionic radii of the atoms used to calculate Goldschmidt (t) and new (τ_{new}) tolerance factors. Ionic radii that could not be found in previous reports is interpolated.

Atom	Ionic radius (Å)	Oxidation (nA)	Coordination number
Li	1.25	1	12
Na	1.39	1	12
K	1.64	1	12
Rb	1.72	1	12
Cs	1.88	1	12
Nb	0.64	5	6
O	1.40	−2	6

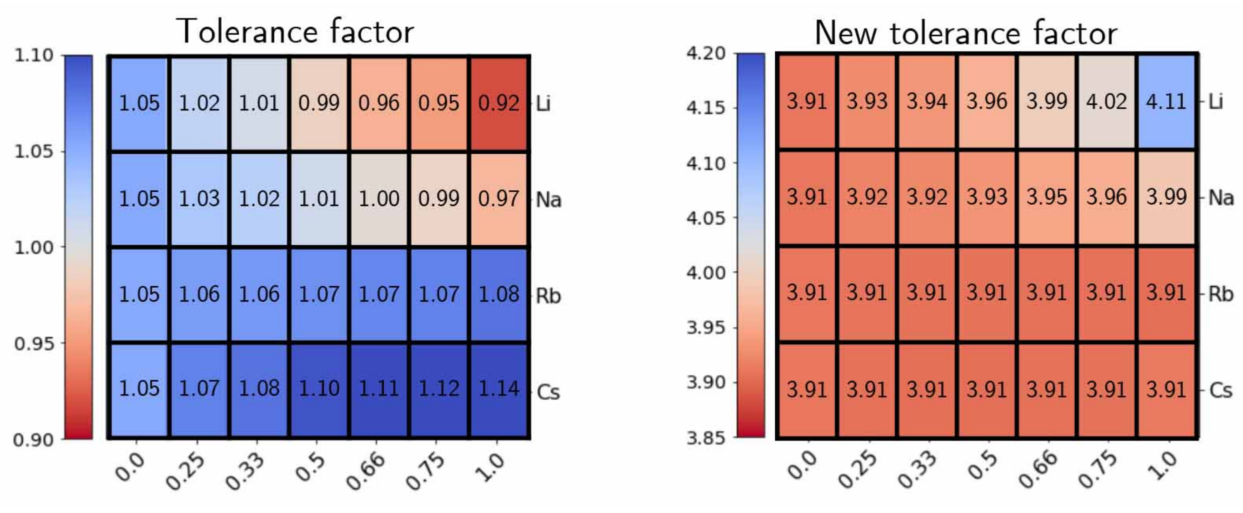

FIGURE S1. The values of the tolerance factor and the new tolerance factor are shown. As noted, almost all structures and compositions are within the boundary of the perovskite forming.

Generally, the introduction of cations with varying ionic radii into the $KNbO_3$ results in lattice distortions, which is linked directly to the formability of the structures. Hence to assess the possibility of solid solution perovskites forming with regard to different size

cations (radius $r_{\mathrm{Li}} < r_{\mathrm{Na}} < r_{\mathrm{K}} < r_{\mathrm{Rb}} < r_{\mathrm{Cs}}$), we have investigated the Goldschmidt tolerance factor [7] as well as a recently proposed the new tolerance factor [8], employing the Shannon ionic radii [9].

Goldschmidt tolerance factor[7], defined as the following:

$$t = \frac{r_{\mathrm{A}} + r_{\mathrm{O}}}{\sqrt{2}\,(r_{\mathrm{B}} + r_{\mathrm{O}})} \tag{S2}$$

where $\mathrm{r_A}$, $\mathrm{r_B}$, and $\mathrm{r_O}$ are the ionic radii of A, B, and O ions, respectively. Empirical results from various data suggest that a perovskite is likely to form in the range of $0.76 < t < 1.11$ [10]. Here, we note that coordination number of 12 and 6 are used for r_{A} and r_{B} respectively. Interpolated values of the radii are used for unobtainable radii data corresponding to the correct coordination numbers. Prior papers also follow the same process as in the Reference 11 and are tabulated in the Table S4. We also note that an arithmetic average of the two cations radius is used in case of the A-site radii (r_{A}) as the following equation:

$$r_{\mathrm{A'}} = (1 - x) \cdot r_{\mathrm{K}} + x \cdot r_{\mathrm{A}} \tag{S3}$$

Form this classic descriptor, the new tolerance factor is suggested based on SISSO [8] to predict the stability. For this newly proposed factor, the likelihood of solid solution being a perovskite increases as t decreases. While $t = 4.18$ being the absolute limit for a perovskite to be formed. It is calculated using the following equation:

$$\tau = \frac{r_X}{r_{\mathrm{B}}} - n_{\mathrm{A}} \left(n_{\mathrm{A}} - \frac{r_{\mathrm{A}}/r_{\mathrm{B}}}{\ln\left(r_{\mathrm{A}}/r_{\mathrm{B}}\right)} \right) \tag{S4}$$

where r_{A}, r_{B}, and r_{O}, are the ionic radii of A, B, O ions respectively and n_{A} is the oxidation state of A.

S4. SOLID-SOLUTION THERMODYNAMICS

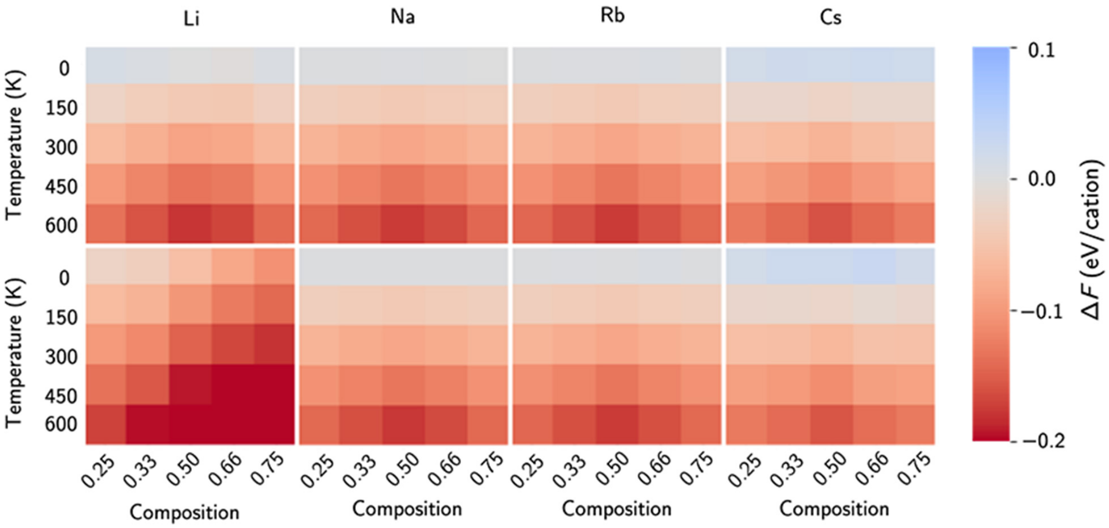

FIGURE S2. Helmholtz free energy for mixing for tetragonal and orthorhombic $K_{1-x}A_xNbO_3$ in the first and second row, respectively. Here, the skyblue colors represent a positive value meaning that the solution is hard to mix, while the red represents a negative value meaning the mixability is high.

To assess the thermodynamic stability of $K_{1-x}A_xNbO_3$ cation solid solution (A = Li, Na, Rb, Cs), we calculate the Helmholtz free energy of mixing for each composition according to

$$\Delta F = \Delta U - T\Delta S \quad , \tag{S5}$$

where ΔU and ΔS are the internal energy and entropy of mixing and T is the absolute temperature. The internal energy of mixing is then calculated using

$$\Delta U = E_{\mathrm{K}_{1-x}\mathrm{A}_x\mathrm{NbO}_3} - (1-x)E_{\mathrm{KNbO}_3} - xE_{\mathrm{ANbO}_3} \quad , \tag{S6}$$

where $E_{\mathrm{K}_{1-x}\mathrm{A}_x\mathrm{NbO}_3}$, E_{KNbO_3}, and E_{ANbO_3} are the total energies of $K_{1-x}A_xNbO_3$, $KNbO_3$, and $ANbO_3$ (A = Li, Na, Rb, and Cs), respectively. The configurational entropy is calculated by the approximated entropy of ideal mixing according to

$$\Delta S = -k_{\mathrm{B}}[x \ln x + (1-x)\ln(1-x)] \quad , \tag{S7}$$

where k_{B} is the Boltzmann constant.

To understand thermodynamic stability of constructed solid solution models [12], based on the optimized structures, we calculated the Helmholtz free energy of mixing for 0 K, 150 K, 300 K, 450 K, and 600 K following equation S5. The calculated Helmholtz free energy is plotted as a function of temperature in Figure S2. At 0 K, the entropy of mixing (ΔS) is neglected because T = 0 K, resulting in most of the considered solid solutions depicting a positive value, indicating that the solid solutions prefer to phase separate into the constituents.

However, as temperature increases, the entropy of mixing affects the Helmholtz free energy. This results in the stability of solids increasing gradually as the temperature increases. At room temperature of 300 K, all solid solutions of $K_{1-x}A_xNbO_3$ (A = Li, Na, Rb, Cs) are calculated to be more stable than the end member phases, which lends support to the possibility of forming the generated perovskite $K_{1-x}A_xNbO_3$ solid solution.

Interestingly, one outlining trend is the $K_{1-x}Li_xNbO_3$ orthorhombic phase. While all other solid solutions tend to show the highest stability at $x = 0.5$ in 600 K, the $K_{1-x}Li_xNbO_3$ orthorhombic phase shows a different result, increasing gradually as the composition increases over the $x = 0.5$. Also, the lithium mixed solid solution results in a negative value even when T = 0 K. This was largely attributed to the influence of the off-center Li ion due to ion-radius misfit with K ion [13]. This dependence has also been observed for other $K_{1-x}Li_xTaO_3$ systems, where the small Li ions take off-center positions with a displacement as large as a quarter of the lattice constant [14, 15]. This is further corroborated in our spontaneous polarization analysis which will be described and discussed in a later section.

S5. STRUCTURAL ANALYSIS

Based on these SQSs, we performed constrained optimization calculations and represent the lattice constants and volume changes in Figure S3 and Figure 1c in the main text, respectively. Additionally, the vectorial distortion of Nb in NbO_6 octahedra are investigated with Bond-elongation following Reference16 and represented in Figure S5

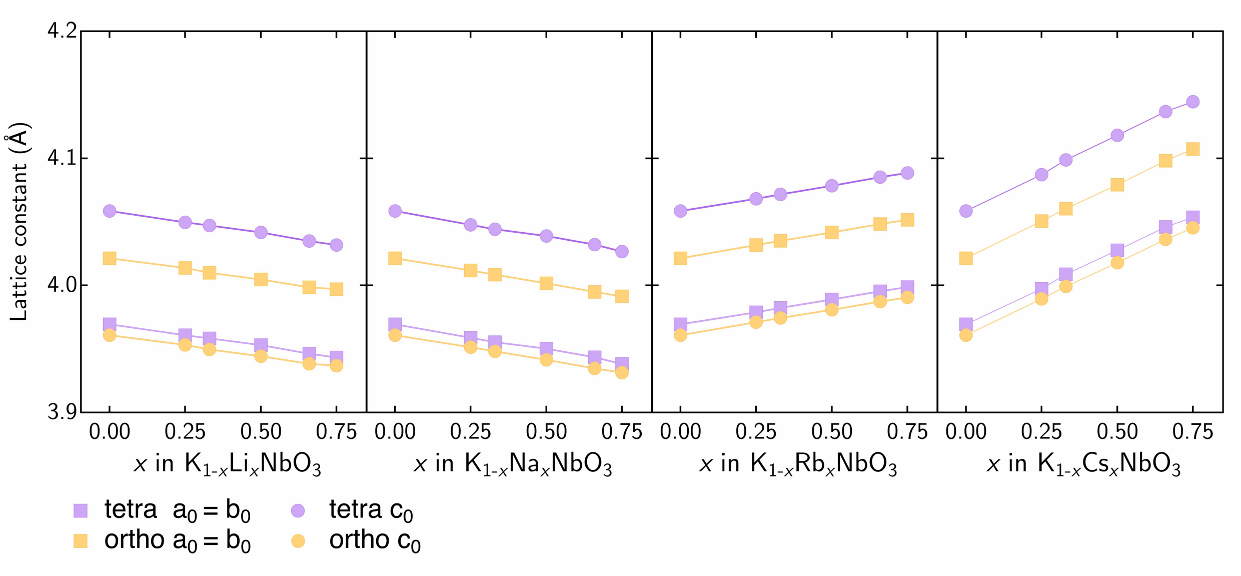

FIGURE S3. DFT-calculated lattice parameters (a_0, b_0, and c_0). All markers are calculated values, between which the dotted lines are fitted to show the trend in the changing values with increasing x. From this figure we note that our SQS generated structures follow the Vegard's law adequately. Show the generated structures are random alloys.

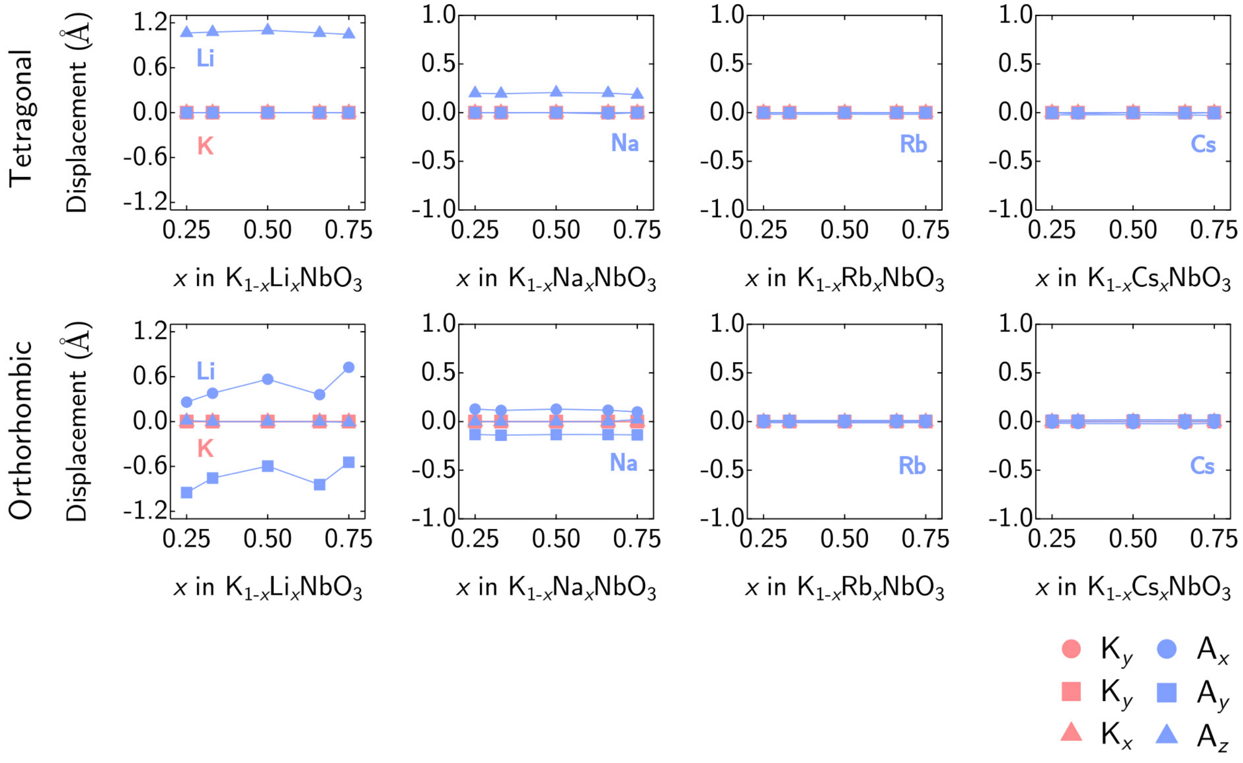

FIGURE S4. The figure shows the A-site displacement of each structure as a function of composition. The top line shows the displacement of tetragonal while the bottom line depicts the orthorhombic data. The red markers represent the K atoms of the A-site while the blue markers represent the A-site substitutes. The different marker shapes depict each direction of x, y, or z displacement.

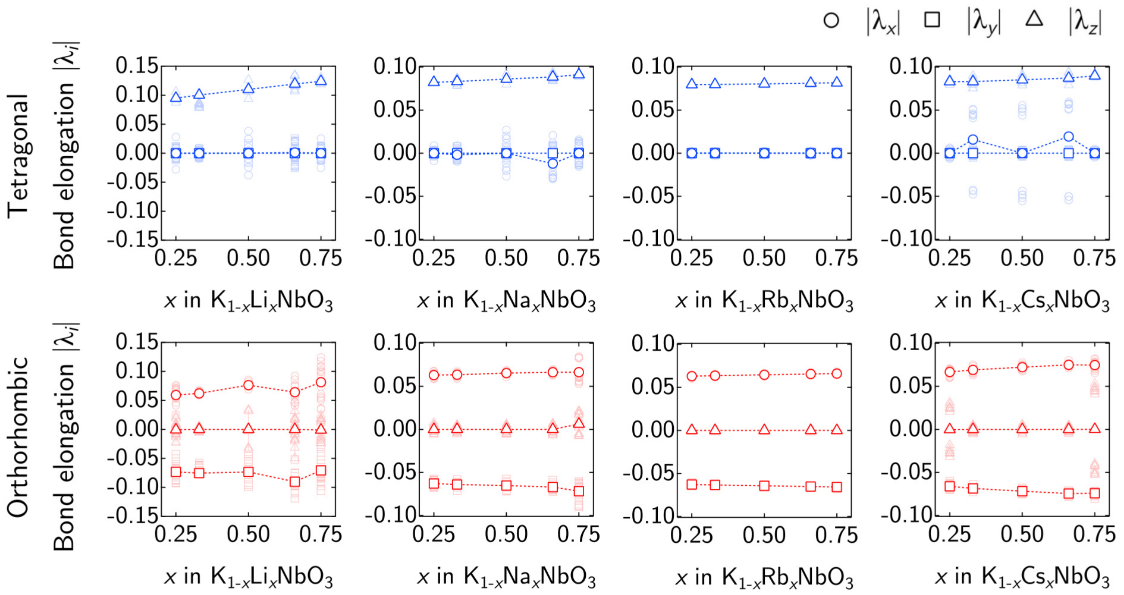

FIGURE S5. Bond elongation index (BEI) results of the tetragonal (top), and orthorhombic (bottom) are plotted as a function of composition. The blurred markers represent each octahedron while the makers connected with the lines are the averaged trend. The different marker shapes depict each direction of x, y, or z.

To quantitatively determine the extent of intra-octahedral distortion (i) within a single polyhedron for each cartesian direction in a given crystal structure, one can define a bond-length-based index (i.e., the bond elongation index, $\overline{\lambda}_i$) as a generic measurement index for polyhedral distortions. Mathematically, the bond elongation index, $\overline{\lambda}_i$ can be expressed as

$$\left[\overline{\lambda}_x, \overline{\lambda}_y, \overline{\lambda}_z\right] = \frac{1}{n}\left[\sum_{i=1}^{n}\frac{\Delta x_i}{l_0}, \sum_{i=1}^{n}\frac{\Delta y_i}{l_0}, \sum_{i=1}^{n}\frac{\Delta z_i}{l_0}\right] \quad , \tag{S8}$$

where Δx_i, Δy_i, and Δz_i are vector components of $\vec{l_i}$, which is a vector from i atom at polyhedron vertex to the center of the polyhedron, in the formal x, y, and z Cartesian axes.

S6. PEARSON CORRELATIONS COEFFICIENTS (PCC)

TABLE S4. The list of site-averaged primary feature classes ($\overline{\mathrm{PF}}$s) along with the abbreviations of each feature and the calculated Pearson correlation coefficient (PCCs) of $\overline{\mathrm{PF}}$s against $\widehat{P}$.

$\overline{\mathrm{PF}}$ (Abb.)	PCC
Electron Affinity (EA)	0.95
Atomic radii (r)	−0.88
Electronegativity (χ)	0.88
Shannon Radii (R)	−0.84
Ionization Energy (IE)	0.83
Polarizability (α)	−0.78
Atomic Mass (M)	−0.68

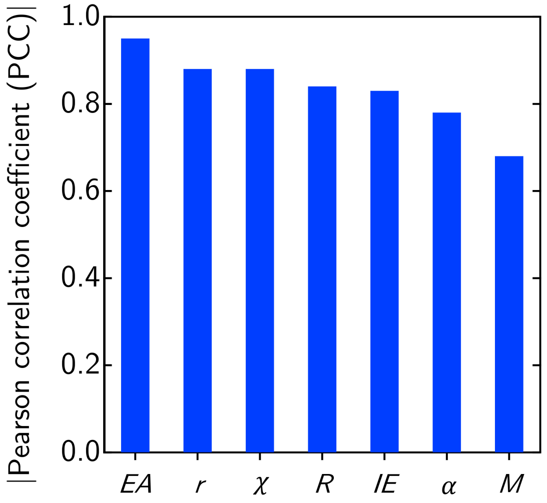

FIGURE S6. The plot of calculated Pearson correlation coefficient (PCCs) of $\overline{\mathrm{PF}}$s against $\widehat{P}$. Absolute values of PCCs are reported.

S7. GENERATION OF VALIDATION SETS FOR THE BINARY $K_{1-x}A_xNBO_3$ ALLOYS

Data augmentation is a technique used to increase the numbers of data via modifying existing data or newly creating synthetic data from existing data. An important deviation from the actual data augmentation method is that while most data augmentation is actually carried out for increasing the training size in hopes of preventing overfitting, we have utilized the interpolated data as a test set to assess the accuracy of the SISSO generated descriptors for the binary $K_{1-x}A_xNbO_3$ alloys only.

Hence, to evaluate the performance of the identified SISSO descriptor based on the 42 DFT data points, an additional 2,004 interpolated data points per phase (by assuming an almost linear behavior – *Vegard-like* (VL) relation – between the DFT data points in Figure 2 in the main text) have been generated using data augmentation concept. Specifically, we have interpolated the A-site boosting ($\widehat{P}$), in the 0.25 0.75 composition of $K_{1-x}A_xNbO_3$ cation solid solution (A = Li, Na, Rb, Cs), resulting in 2004 interpolated data points. For the interpolation of the data points based on the VL relation, the P_A and P_S are separately fitted for $y = ax$ and $y = ax + b$ respectively. Here we note that the P_A is fitted to a linear model with no intercept, in order to enforce the physical rule of KNO having no A-site polarization. From the fitted results, the interpolated data points are divided to calculate $\widehat{P}$ since, $\widehat{P} = \dfrac{P_\text{A}}{P_\text{S}}$.

S8. DATA SAMPLING

Data sampling for the test of number of training data points (N_t) converging is done by two different methods, a supervised method to ensuring linearity, and an unsupervised method randomizing the data points. For the supervised method, two factors are considered. First equal distribution of points in different classes. Here, we refer to classes as a different substitution. To elaborate, we have four different substitutions Li, Na, Rb, and Cs. We refer to each of these for substitution as a class, and when we are selecting the data points, we enforce that each data selected from each class always is equal to each other when we are sampling the data the in a supervised way. Second, equal spacing between the compositions is also considered. While for a unsupervised sample of data, random data points are selected with 100 iterations.

PRIMARY FEATURES

TABLE S5. The averaged radii, electronegativity, electron affinity of each composition according to substitute.

Type		Primary Feature		
Subsitute	Composition	$\bar{r}_{\mathrm{A}}$	$\overline{\chi}_{\mathrm{A}}$	$\overline{EA}_{\mathrm{A}}$
–	0.00	2.20	0.820	48.40
Li	0.25	2.01	0.860	51.20
Li	0.33	1.95	0.873	52.10
Li	0.50	1.83	0.900	54.00
Li	0.66	1.71	0.926	55.79
Li	0.75	1.64	0.940	56.80
Na	0.25	2.10	0.848	49.50
Na	0.33	2.07	0.856	49.85
Na	0.50	2.00	0.880	50.60
Na	0.66	1.94	0.893	51.30
Na	0.75	1.90	0.903	51.70
Rb	0.25	2.24	0.820	48.03
Rb	0.33	2.25	0.820	47.91
Rb	0.50	2.28	0.820	47.65
Rb	0.66	2.23	0.820	47.41
Rb	0.75	2.31	0.820	47.26
Cs	0.25	2.30	0.813	47.68
Cs	0.33	2.33	0.810	47.44
Cs	0.50	2.40	0.805	46.95
Cs	0.66	2.46	0.800	46.49
Cs	0.75	2.50	0.798	46.23

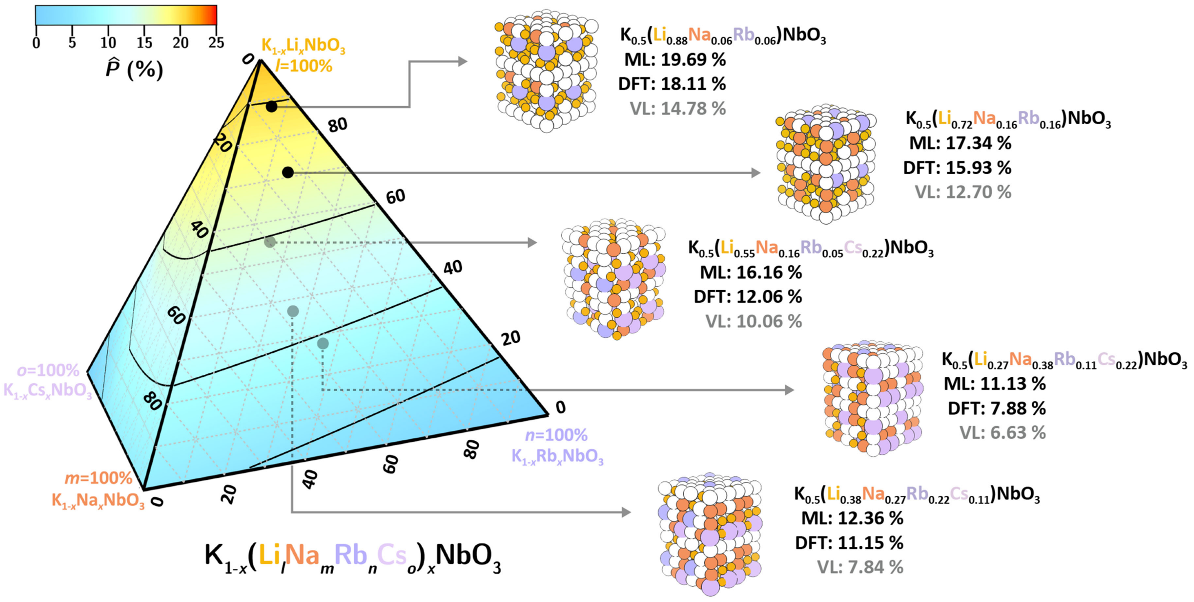

FIGURE S7. Predicted $\widehat{P}$ values for the multicomponent orthorhombic penternary $K_{1-x}(Li_lNa_mRb_nCs_o)_xNbO_3$ alloy. Here, plots are presented as tetrahedron-shaped quarternary $\widehat{P}$ diagrams for a particular fixed K concentration (x = 0.5). SQS-DFPT $\widehat{P}$ values (and their corresponding VL-estimated values) for five alloys are also listed to assist in the validation of the SISSO R_1 predicted $\widehat{P}$ values.

OPTIMIZED SISSO MODEL: DESCRIPTORS

Considering the 2524st ranked SISSO regression model (R_{2524}), the R_{2524} SISSO descriptors (D^1_{2524} and D^2_{2524}) are:

$$D^1_{2524} : \exp\left(\frac{EA_{\mathrm{Nb}}}{\overline{EA}_{\mathrm{A}}}\right) \cdot \left(\frac{\overline{r}_{\mathrm{A}}}{r_{\mathrm{Nb}}} + \frac{EA_{\mathrm{O}}}{\overline{EA}_{\mathrm{A}}}\right)^{-1} \quad , \tag{S9}$$

$$D^2_{2524} : \left|\overline{r}_{\mathrm{A}} \cdot \left(\frac{EA_{\mathrm{Nb}}}{\overline{EA}_{\mathrm{A}}}\right) - \left(\frac{\overline{r}_{\mathrm{A}} \cdot EA_{\mathrm{Nb}}}{\exp(\overline{\chi}_{\mathrm{A}})}\right)\right| \quad . \tag{S10}$$

The fitting coefficients of $\widehat{P}$ for the tetragonal and orthorhombic $KNbO_3$-based alloys can be predicted using:

$$\widehat{P}_{\mathrm{tetra}} = -252.46 D^1_{2524} + 1.53 D^2_{2524} \quad , \tag{S11}$$

and

$$\widehat{P}_{\mathrm{ortho}} = -206.97 D^1_{2524} + 1.55 D^2_{2524} \quad , \tag{S12}$$

respectively.

TABLE S6. The experimental spontaneous polarization of various lead-free ferroelectric material. Here we note that the symbols R, O, and T are used to denote the rhombohedral, orthorhombic, and tetragonal phases, respectively.

Material	P_s (μC/cm^2)	Reference
$Bi_4Ti_3O_{12}$	45.35	17
HfO_2	44.96	18
$KNbO_3$ (R)	42.00	19
$KNbO_3$ (O)	41.00	20
$KNbO_3$ (T)	37.00	21
$BaTiO_3$ (R)	33.00	22
$BaTiO_3$ (O)	30.00	22
$SrBi_4Ti_4O_{15}$	27.83	23
$BaTiO_3$ (T)	27.00	22
$SrBi_2Ta_2O_9$	18.88	24
$BaBi_4Ti_5O_{15}$	13.82	25
$Ba_2Bi_4Ti_5O_{18}$	10.51	25

[1] F. Schmidt, M. Landmann, E. Rauls, N. Argiolas, S. Sanna, A. Schindlmayr. Consistent Atomic Geometries and Electronic Structure of Five Phases of Potassium Niobate from Density-Functional Theory. *Adv. Mater. Sci. Eng.* **2017**, *2017*, 3981317.

[2] S. Simsek, H. Koc, V. A. Trepakov, A. M. Mamedov, E. Ozbay. Electron Spectroscopy and the Electronic Structure of $KNbO_3$: First Principle Calculations. *Ferroelectrics* **2014**, *461*, 99–105.

[3] M. Fontana, G. Metrat, J. Servoin, F. Gervais. Infrared Spectroscopy in $KNbO_3$ Through the Successive Ferroelectric Phase Transitions. *J. Phys. C: Solid State Phys.* **2000**, *17*, 483.

[4] A. Zunger, S.-H. Wei, L. G. Ferreira, J. E. Bernard. Special Quasirandom Structures. *Phys. Rev. Lett.* **1990**, *65*, 353–356.

[5] S.-H. Wei, L. G. Ferreira, J. E. Bernard, A. Zunger. Electronic Properties of Random Alloys: Special Quasirandom Structures. *Phys. Rev. B* **1990**, *42*, 9622–9649.

[6] M. Ångqvist, W. A. Muñoz, J. M. Rahm, E. Fransson, C. Durniak, P. Rozyczko, T. H. Rod, P. Erhart. ICET – A Python Library for Constructing and Sampling Alloy Cluster Expansions. *Adv. Theory Simul.* **2019**, *2*, 1900015.

[7] V. M. Goldschmidt. Die Gesetze der Krystallochemie. *Naturwissenschaften* **1926**, *14*, 477–485.

[8] C. Bartel, C. Sutton, B. R. Goldsmith, R. Ouyang, C. B. Musgrave, L. M. Ghiringhelli, M. Scheffler. New Tolerance Factor to Predict the Stability of Perovskite Oxides and Halides. *Sci. Adv.* **2019**, *5*, eaav0693.

[9] R. D. Shannon. Revised Effective Ionic Radii and Systematic Studies of Interatomic Distances in Halides and Chalcogenides. *Acta Cryst. A* **1976**, *32*, 751–767.

[10] M. A. Green, A. Ho-Baillie, H. J. Snaith. The Emergence of Perovskite Solar Cells. *Nat. Photonics* **2014**, *8*, 506–514.

[11] R. D. Shannon, R. X. Fischer. Empirical Electronic Polarizabilities in Oxides, Hydroxides, Oxyfluorides, and Oxychlorides. *Phys. Rev. B* **2006**, *73*, 235111.

[12] Y. K. Jung, J.-H. Lee, A. Walsh, A. Soon. Influence of Rb/Cs Cation-Exchange on Inorganic Sn Halide Perovskites: From Chemical Structure to Physical Properties. *Chem. Mater.* **2017**, *29*, 3181–3188.

[13] R. Machado, M. Sepliarsky, M. G. Stachiotti. Off-Center Impurities in a Robust Ferroelectric

Material: Case of Li in $KNbO_3$. *Phys. Rev. B* **2012**, *86*, 094118.

[14] R. I. Eglitis, A. V. Postnikov, G. Borstel. Semiempirical Hartree-Fock Calculations for Pure and Li-doped $KTaO_3$. *Phys. Rev. B* **1997**, *55*, 12976.

[15] M. G. Stachiotti, R. L. Migoni. Lattice Polarisation Around Off-Centre Li in $Li_xK_{1-x}TaO_3$. *J. Phys.: Condens. Matter* **1990**, *2*, 4341.

[16] W. Hwang, J.-H. Lee, A. Soon. Enhanced Polarization in Epitaxially Strained Monoclinic Potassium Niobate for Lead-Free Electromechanical Applications. *J. Mater. Chem. C* **2021**, *9*, 13420–13431.

[17] Y. Noguchi, T. Matsumoto, M. Miyayama. Impact of Defect Control on the Polarization Properties in $Bi_4Ti_3O_{12}$ Ferroelectric Single Crystals. *Jpn. J. Appl. Phys.* **2005**, *44*, L570.

[18] T. Shimizu, K. Katayama, T. Kiguchi, A. Akama, T. J. Konno, O. Sakata, H. Funakubo. The Demonstration of Significant Ferroelectricity in Epitaxial Y-doped HfO_2 Film. *Sci. Rep.* **2016**, *6*, 1–8.

[19] A. W. Hewat. Cubic-Tetragonal-Orthorhombic-Rhombohedral Ferroelectric Transitions in Perovskite Potassium Niobate: Neutron Powder Profile Refinement of the Structures. *J. Phys. C: Solid State Phys.* **1973**, *6*, 2559.

[20] F. M. Michel-Calendini, M. Peltier, F. Micheron. Electron Paramagnetic Resonance of Fe3+ in Orthorhombic $KNbO_3$. *Solid State Commun.* **1980**, *33*, 145–150.

[21] W. Kleemann, F. J. Schäfer, M. D. Fontana. Crystal Optical Studies of Spontaneous and Precursor Polarization in $KNbO_3$. *Phys. Rev. B* **1984**, *30*, 1148.

[22] H. H. Wieder. Electrical Behavior of Barium Titanatge Single Crystals at Low Temperatures. *Phys. Rev* **1955**, *99*, 1161.

[23] H. Irie, M. Miyayama. Dielectric and Ferroelectric Properties of $SrBi_4Ti_4O_{15}$ Single Crystals. *Appl. Phys. Lett.* **2001**, *79*, 251–253.

[24] H. Amorin, V. V. Shvartsman, A. L. Kholkin, M. E. V. Costa. Ferroelectric and Dielectric Anisotropy in High-Quality $SrBi_2Ta_2O_9$ Single Crystals. *Appl. Phys. Lett.* **2004**, *85*, 5667–5669.

[25] H. Irie, M. Miyayama, T. Kudo. Structure Dependence of Ferroelectric Properties of Bismuth Layer-Structured Ferroelectric Single Crystals. *J. Appl. Phys.* **2001**, *90*, 4089–4094.
